# CSF biomarkers of neuroinflammation are associated with regional atrophy

**DOI:** 10.1007/s00415-025-13564-5

**Published:** 2025-12-24

**Authors:** Serap Özlü, Martin Dyrba, Alice Grazia, Frederic Brosseron, Katharina Buerger, Peter Dechent, Emrah Düzel, Michael Ewers, Klaus Fliessbach, Wenzel Glanz, Niels Hansen, Julian Hellmann-Regen, Stefan Hetzer, Daniel Janowitz, Ingo Kilimann, Marie Kronmüller, Christoph Laske, Falk Lüsebrink, David Mengel, Robert Perneczky, Oliver Peters, Josef Priller, Alfredo Ramirez, Boris-Stephan Rauchmann, Ayda Rostamzadeh, Anja Schneider, Sebastian Sodenkamp, Annika Spottke, Eike Jakob Spruth, Matthis Synofzik, Jens Wiltfang, Michael T Heneka, Frank Jessen, Stefan Teipel

**Affiliations:** 1 German Center for Neurodegenerative Diseases (DZNE), Rostock, Germany; 2 Department of Psychosomatic Medicine, Rostock University Medical Center, Rostock, Germany; 3 German Center for Neurodegenerative Diseases (DZNE), Bonn, Germany; 4 German Center for Neurodegenerative Diseases (DZNE), Munich, Germany; 5 Institute for Stroke and Dementia Research (ISD), University Hospital, LMU Munich, Munich, Germany; 6 MR-Research in Neurosciences, Department of Cognitive Neurology, University Medical Center Goettingen, Goettingen, Germany; 7 German Center for Neurodegenerative Diseases (DZNE), Magdeburg, Germany; 8 Institute of Cognitive Neurology and Dementia Research (IKND), Otto-Von-Guericke University, Magdeburg, Germany; 9 Department of Old Age Psychiatry and Cognitive Disorders, University Hospital Bonn and University of Bonn, Bonn, Germany; 10 Department of Psychiatry and Psychotherapy, University Medical Center Goettingen, University of Goettingen, Goettingen, Germany; 11 German Center for Neurodegenerative Diseases (DZNE), Berlin, Germany; 12 Department of Psychiatry and Neurosciences, Charité Universitätsmedizin Berlin, Berlin, Germany; 13 ECRC Experimental and Clinical Research Center, Charité Universitätsmedizin Berlin, Berlin, Germany; 14 Berlin Center for Advanced Neuroimaging, Charité-Universitätsmedizin Berlin, Berlin, Germany; 15 German Center for Neurodegenerative Diseases (DZNE), Tübingen, Germany; 16 Section for Dementia Research, Hertie Institute for Clinical Brain Research and Department of Psychiatry and Psychotherapy, University of Tübingen, Tübingen, Germany; 17 Division of Translational Genomics of Neurodegenerative Diseases, Hertie Institute for Clinical Brain Research and Center of Neurology, University of Tübingen, Tübingen, Germany; 18 Department of Psychiatry and Psychotherapy, University Hospital, LMU Munich, Munich, Germany; 19 Munich Cluster for Systems Neurology (SyNergy) Munich, Munich, Germany; 20 Ageing Epidemiology Research Unit (AGE), School of Public Health, Imperial College London, London, UK; 21 Department of Psychiatry and Psychotherapy, Charité-Universitätsmedizin Berlin, Berlin, Germany; 22 University of Edinburgh and UK DRI, Edinburgh, UK; 23 Department of Psychiatry and Psychotherapy, School of Medicine and Health, Technical University of Munich, and German Center for Mental Health (DZPG), Munich, Germany; 24 Cluster of Excellence on Cellular Stress Responses in Aging-Associated Diseases (CECAD), University of Cologne, Cologne, Germany; 25 Division of Neurogenetics and Molecular Psychiatry, Department of Psychiatry and Psychotherapy, Faculty of Medicine and University Hospital Cologne, University of Cologne, Cologne, Germany; 26 Department of Psychiatry &, Glenn Biggs Institute for Alzheimer’s and Neurodegenerative Diseases, San Antonio, TX USA; 27 Sheffield Institute for Translational Neuroscience (SITraN), University of Sheffield, Sheffield, UK; 28 Department of Neuroradiology, University Hospital LMU, Munich, Germany; 29 Department of Psychiatry, Medical Faculty, University of Cologne, Cologne, Germany; 30 Department of Psychiatry and Psychotherapy, University of Tübingen, Tübingen, Germany; 31 Clinic for Parkinson’s, Sleep and Movement Disorders, Centre for Neurology, University Hospital Bonn, Bonn, Germany; 32 German Center for Neurodegenerative Diseases (DZNE), Goettingen, Germany; 33 Neurosciences and Signaling Group, Institute of Biomedicine (iBiMED), Department of Medical Sciences, University of Aveiro, Aveiro, Portugal; 34 Luxembourg Centre for Systems Biomedicine, University of Luxembourg, Esch-sur-Alzette, Luxembourg

**Keywords:** Alzheimer’s disease, Neuroinflammation, Biomarker, Hippocampus, Basal forebrain

## Abstract

**Background:**

Neuroinflammation is central to Alzheimer’s disease (AD) pathogenesis, yet its contribution to region‐specific brain atrophy remains unclear. We examined whether cerebrospinal fluid (CSF) biomarkers predict longitudinal atrophy in the hippocampus and basal forebrain and mediate the impact of AD pathology.

**Methods:**

Data from 227 DELCODE participants with baseline CSF measures and longitudinal structural MRI were analyzed. Four latent factors (synaptic, microglia, chemokine/cytokine, complement) were derived to capture shared variance across biomarkers. Latent factors represent unobserved biological domains inferred from related CSF markers. In addition, four single biomarkers (neurogranin, sTREM2, YKL-40, ferritin) were tested separately. Regional atrophy rates were estimated using linear mixed-effects models including biomarker × time, A/T classification, diagnosis, and covariates (age, sex, education, ApoE-ε4). Individual slopes were then entered into mediation models.

**Results:**

Higher synaptic latent factor (*β* = − 0.019, pFDR = 0.021) and YKL-40 (*β* = − 0.020, pFDR = 0.025) significantly predicted hippocampal atrophy. Only these two markers remained significant after correction for multiple comparisons. Mediation analyses revealed significant indirect effects of the synaptic latent factor and YKL-40 on hippocampal atrophy across all A/T groups. No biomarker was associated with basal forebrain atrophy (pFDR > 0.05).

**Conclusions:**

Latent factors captured shared biological variance across related biomarkers and provided a more robust representation of underlying biological domains than single biomarkers. This approach identified synaptic dysfunction and astroglial activation as key links between AD pathology and hippocampal neurodegeneration. These findings highlight synaptic and glial pathways as promising targets for disease-modifying interventions.

**Supplementary Information:**

The online version contains supplementary material available at 10.1007/s00415-025-13564-5.

## Introduction

Neuroinflammation has emerged as a core pathological feature of Alzheimer’s disease (AD), alongside amyloid-β (Aβ) and tau aggregation, progressive brain atrophy, and cognitive decline [1–3]. However, the role of cerebrospinal fluid (CSF) neuroinflammatory markers in predicting region‐specific atrophy over time has yet to be firmly established. Demonstrating these links could reveal the relationship between immune processes and neurodegeneration and help to identify new therapeutic targets. In addition, combining inflammatory markers with established CSF measures of Aβ and tau may improve predictions of regional atrophy patterns [3].

The hippocampus and the basal forebrain are among the most affected regions in AD [4–6]. The hippocampus supports episodic memory formation [4], and its atrophy is an established biomarker of disease progression [7–9]. The cholinergic basal forebrain, which releases acetylcholine to widespread cortical and hippocampal areas, supports attention, learning, and memory [6, 10]. In AD, these neurons undergo progressive degeneration, resulting in a decrease in cholinergic function [11, 12]. This decrease correlates with cognitive impairment and greater clinical severity [10–12]. Despite the progressive atrophy in both regions [13, 14], the role of neuroinflammation remains unclear.

Microglial activation and synaptic degeneration in the hippocampus have been implicated in cognitive impairment [15, 16]. However, longitudinal findings from individual CSF biomarkers reflecting these processes have been inconsistent. For instance, elevated CSF levels of neurogranin, a postsynaptic plasticity protein, have been associated with faster hippocampal atrophy and cognitive decline in some cohorts [17], whereas other studies have reported no such associations [18, 19]. CSF YKL-40 (also known as chitinase-3-like protein 1 (CHI3L1)), a glycoprotein secreted primarily by activated astrocytes [20], has been linked to disease progression, cognitive decline, and regional atrophy [21, 22], though results vary across cohorts [18]. The findings for CSF sTREM2 (soluble triggering receptor expressed on myeloid cells 2), a marker of microglial activation, are similarly heterogeneous, with studies reporting stage and context-dependent associations with cognition and neurodegeneration [23–26]. These discrepancies might be due to heterogeneity in study design, disease stage, and the limitations of using single markers to reflect complex inflammatory processes.

To address these limitations, we applied latent factor modeling using confirmatory factor analysis (CFA). A latent factor is an unobserved variable inferred from multiple related biomarkers that reflect a shared underlying biological domain. This approach combines biomarkers into a single composite score that captures their shared variance, reduces measurement error, and provides a more reliable estimate of the underlying construct [27, 28].

Building on our previous work, we used four predefined latent factors representing distinct biological domains relevant to AD pathology: synaptic, microglial, chemokine/cytokine, and complement [28]. These domains reflect key components of neuroinflammatory and synaptic pathology, including synaptic dysfunction, microglial activation, chemokine/cytokine signaling, and complement activity [28]. Using these biologically grounded constructs, we aimed to understand how these factors relate to longitudinal atrophy in the hippocampus and basal forebrain and whether they mediate the effects of AD pathology. In addition to the latent constructs, we tested four individual CSF biomarkers—neurogranin, sTREM2, ferritin, and YKL-40—that have shown heterogeneous associations with the hippocampal and basal forebrain atrophy in previous studies [17–26, 29–32]. Examining these markers individually enabled us to assess whether they exhibit region-specific effects that might not be fully reflected by the latent factors.

## Methods

### Study design

DELCODE is an ongoing, longitudinal, multicenter study that was initiated in 2014 in Germany. The study design and methodology have been described in detail by Jessen et al. (2018) [33] and include a comprehensive diagnostic work-up consisting of clinical and neuropsychological assessments, neuroimaging, and annual follow-up visits. The study protocol was approved by the local institutional review boards and ethics committees of all participating sites. The research is conducted in accordance with the Declaration of Helsinki. All participants provided written informed consent prior to participation.

### Participants

The DELCODE study includes individuals with subjective cognitive decline (SCD), mild cognitive impairment (MCI), and AD dementia, as well as a control group and cognitively healthy relatives of AD patients [33]. Healthy controls (HC) were defined as individuals with no cognitive complaints, performing within normal ranges on cognitive tests, and without a history of neurological or psychiatric illness [33]. First‐degree relatives of AD patients, here also included in the HC group, were similarly cognitively healthy but had at least one immediate family member diagnosed with AD [33]. SCD was defined in accordance with the criteria set by the SCD Initiative Working Group [34]. The criteria for self-reported cognitive decline in the absence of objective cognitive impairment was assessed using the Consortium to Establish a Registry for Alzheimer’s Disease (CERAD) test battery [34]. Diagnoses of MCI and AD were based on the criteria established by the National Institute on Aging-Alzheimer’s Association (NIA-AA) workgroup [35–37]. Because the syndromic classifications may not fully reflect the biological heterogeneity of AD, we applied a biomarker-based A/T (Amyloid beta/Tau) classification in addition to clinical diagnosis to represent disease pathology.

### MRI acquisition and preprocessing

The MRI data was acquired using various Siemens 3 T scanners [33] following a harmonized study protocol. Preprocessing of T1-weighted structural MRI scans was conducted in MATLAB (R2020a) using SPM12 (revision 7487) and the CAT12 toolbox. Longitudinal scans were spatially normalized to the MNI reference space with the CAT12.8 longitudinal segmentation pipeline (revision 1872) using the DARTEL algorithm [38]. Hippocampal gray matter volumes were extracted via the Harvard–Oxford atlas [39], thresholded at a 0.5 probability level. A basal forebrain mask, previously developed by our group [40], was applied to derive regional basal forebrain volumes.

### CSF biomarkers

Data of CSF biomarkers were centrally analyzed at the DZNE Biorepository Facility in Bonn. CSF Aβ42 and pTau181 (phosphorylated tau 181) concentrations were measured using V-PLEX Aβ Peptide Panel 1 (6E10) Kit (K15200E) and Innotest Phospho-Tau(181P) (81,581; Fujirebio Germany GmbH, Hannover, Germany) respectively. All procedures were carried out in accordance with the vendor protocols [33]. The panel of biomarkers was determined by commercially available assays [41–43]. Cutoff values for abnormal CSF biomarkers were defined as Aβ42/40 < 0.09 and pTau181 > 57 pg/ml [33].

### Latent factor modeling with Bayesian CFA

In the Bayesian CFA, CSF markers were assigned a priori to one of four latent factors: synaptic, microglial, chemokine/cytokine, or complement (Table 1). The predefined structure was then tested against the data to assess whether the factor loadings fit the hypothesized model. This allowed each latent score to represent a distinct biological domain [28]. Briefly, the synaptic factor includes markers of dendritic and synaptic degeneration as well as an indicator of iron dysregulation. Higher values reflect greater synaptic dysfunction [44]. The microglial factor comprises proteins associated with microglial activation. These proteins rise as microglia respond to accumulating Aβ and tau [45]. The chemokine/cytokine factor consists of proteins that coordinate intercellular communication and regulation of macrophages [46]. Lastly, the complement factor is involved in the innate immune system and includes components of the complement pathway [47].

**Table 1:** Latent factors and their constituent CSF biomarkers

Latent factors	Included markers
Synaptic	Neurogranin, Ferritin, FABP3
Microglia	sTREM2, YKL-40, MIF-1α, sAXL
Chemokine/cytokine	MCP-1, IP-10, IL-6, IL-18
Complement	C4, Factor B, Factor H

*C4* Complement Component 4, *FABP3* Fatty Acid Binding Protein 3, *IL-6* Interleukin-6, *IL-18* Interleukin-18, *IP-10* Interferon Gamma-Induced Protein 10, *MCP-1* Monocyte Chemoattractant Protein-1, *MIF-1α* Macrophage Migration Inhibitory Factor-1α, *sAXL* Soluble AXL Receptor Tyrosine Kinase, *sTREM2* Soluble Triggering Receptor Expressed on Myeloid Cells 2, *YKL-40* Chitinase-3-Like Protein 1

The synaptic latent factor included CSF biomarkers neurogranin, FABP3 (fatty acid binding protein 3), and ferritin [28]. Among these markers, neurogranin and FABP3 are particularly related to synaptic pathology, with neurogranin representing postsynaptic signaling [44] and FABP3 being associated  with neurodegeneration [48]. Ferritin is a marker of brain iron storage and oxidative stress and has a multi-faceted role in AD [31]. For exploratory purposes, we additionally tested with an alternative synaptic latent factor that excluded ferritin.

### Statistical analysis

Participant characteristics were compared across diagnostic groups using *χ*^2^ tests for categorical variables and Kruskal–Wallis tests for continuous measures. Group differences in baseline CSF markers were examined using ANCOVA models with diagnosis as the between-subject factor, controlling for age and sex. Post hoc pairwise comparisons were conducted with Bonferroni correction for multiple testing. Based on cutoff values, participants were classified into four groups according to their Amyloid beta (A) and Tau (T) biomarker status: A-T-, A-T +, A + T-, and A + T + [49]. This classification represented AD pathology status, and here A-T- was used as the reference group in the models.

We used linear mixed‐effects models to examine whether baseline CSF biomarkers were associated with longitudinal atrophy in the hippocampus and basal forebrain. For each region, the dependent variable was normalized regional volume (adjusted for total intracranial volume (TIV)). Fixed effects included the biomarker of interest, diagnosis (AD, MCI, SCD, HC), and the A/T classification, each interacting with time (years since baseline). Age, sex, education, and ApoE-ε4 carrier status were included as covariates. All continuous variables were standardized (z‐scored) prior to analysis. Random intercepts and slopes for time were specified for each participant to account for individual‐level variation. Models were fit in R (v4.4.2) using the “lmer” function from the *lmerTest* package, and the plots were generated using the *ggplot2* package. The statistical model was formally defined as:

$$ROI \, Volume \, \sim \, mar\ker \, + \, diagnosis \, + \, mar\ker \, \times \, time \, + \, diagnosis \, \times \, time \, + \, A/T \, \times \, time \, + \, A/T \, + \, age \, + \, sex \, + \, education \, + \, ApoE - \varepsilon 4 \, status \, + \, \left( {1 \, + \, time \, | \, Participant \, ID} \right)$$

For sensitivity analysis, the same models were repeated using each of the individual markers that were used to construct the latent factors. False discovery rate (FDR) correction was applied across models to account for multiple comparisons. For each brain region, p-values from all models within the same group (four latent factors and 14 individual markers (four markers of interest and 10 from the panel)) were corrected separately. Next, to test whether neuroinflammation markers mediate the impact of AD pathology on atrophy in the hippocampus and basal forebrain, we first derived individual rates of volume change by fitting linear mixed‐effects models with random intercepts and slopes for years since baseline for each participant. The resulting regional atrophy slopes were compared across diagnostic groups using ANCOVA models with age and sex included as covariates. The slopes were then entered as the outcome in a structural equation mediation analysis. In this model, A/T biomarker status (dummy‐coded) predicted the neuroinflammatory marker in the “a” path, adjusting for ApoE-ε4 carrier status, age, sex, and diagnostic group (dummy‐coded). The “b” path linked the biomarker to regional atrophy slope, controlling for the same covariates. The direct effect (“c”) represented the residual influence of pathology on atrophy independent of marker levels. We computed the indirect effect as “a*b”, and the total effect as the sum of direct and indirect paths (for each A/T group). All continuous variables were z‐scored, and models were estimated using the “sem” function in the *lavaan* package in R (v4.4.2). Mediation analyses were performed only for biomarkers that showed a significant interaction with time (pFDR < 0.05) in the linear mixed-effect models. The mediation model was defined as:

$${\mathrm{Mediator}}: \, Mar\ker \, \sim \, a \, * \, A/T \, + \, ApoE - \varepsilon 4 \, status \, + \, age \, + \, sex \, + \, diagnosis$$

$${\mathrm{Outcome}}: \, ROI \, slope \, \sim \, b \, * \, Mar\ker \, + \, c \, * \, A/T \, + \, ApoE - \varepsilon 4 \, status \, + \, age \, + \, sex \, + \, diagnosis$$

## Results

### Demographics

The demographic and biomarker profiles of the N = 227 participants from the DELCODE cohort with available baseline data of CSF neuroinflammatory markers are presented in Table 2. In the sample, all participants had at least one follow-up MRI scan, with a median follow-up time of 1.5 years (range 0.6–4.9 years; see also Supplementary Table 1).

**Table 2:** Demographic characteristics of the DELCODE sample

Variables	AD	MCI	SCD	HC	*p* value^1^
N	20	49	75	83	
Sex (f/m)	12/8	18/31	35/40	52/31	0.02
Age	73.6(5.9)	72.4(5.6)	71(5.2)	68.2(4.8)	< 0.001
Education	13.9(3.02)	14.2(3.08)	15.02(3.2)	14.3(2.7)	0.25
APOE (ε4 +)	14	17	23	18	< 0.001
MMSE	23.5(3.5)	27.9(1.7)	29.1(0.8)	29.3(0.9)	< 0.001
Amyloid +	20	32	28	26	< 0.001
Tau +	18	30	22	26	< 0.001

Values are presented as mean and standard deviation (SD) in parentheses

*AD* Alzheimer’s disease, *HC* Healthy control, *MCI* Mild cognitive impairment, *MMSE* Mini-Mental State Examination, *SCD* Subjective cognitive decline

^1^p-values obtained from Pearson *χ*^2^ tests for categorical variables and Kruskal–Wallis test for continuous variables

### CSF biomarkers, latent factors, and regions of interest across diagnostic groups

**Fig. 1:**
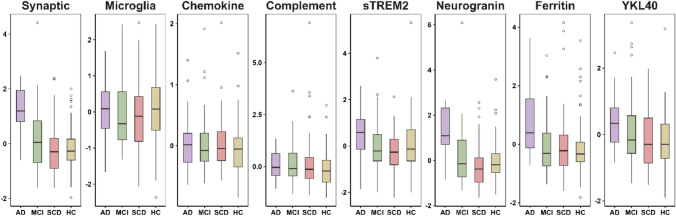
Levels of inflammatory markers across diagnostic groups*.* In each panel, the central line represents the median, the boxes show the 25–75 percentiles, and dots indicate outliers. (*N* = 227, AD = 20, MCI = 49, SCD = 75, HC = 83). *AD* Alzheimer’s disease, *HC* Healthy control, *MCI* Mild cognitive impairment, *SCD* Subjective cognitive decline

Figure 1 shows boxplots of eight *z*-scored CSF measures, including four latent factors (synaptic, microglia, chemokine, complement), and four individual markers (sTREM2, neurogranin, ferritin, YKL-40) stratified by diagnostic group (see also Supplementary Fig. 1 for marker levels across A/T groups). We conducted ANCOVA models for markers across diagnostic groups (AD, MCI, SCD, and HC), controlling for age and sex. Significant group differences were found for synaptic, sTREM2, neurogranin, ferritin, and YKL-40 (*p* < 0.05; see Supplementary Table 2). Following up, pairwise comparisons were performed with Bonferroni correction for multiple testing. Among the latent factors, the AD group showed higher synaptic factor levels compared to the MCI, SCD, and HC groups (p_Bonferroni-adjusted_ < 0.001). Among the individual markers, neurogranin and ferritin levels were higher in AD compared to the other groups (p_Bonferroni-adjusted_ < 0.001 and < 0.05 respectively; see Supplementary Table 3 for all pairwise comparisons).

**Fig. 2:**
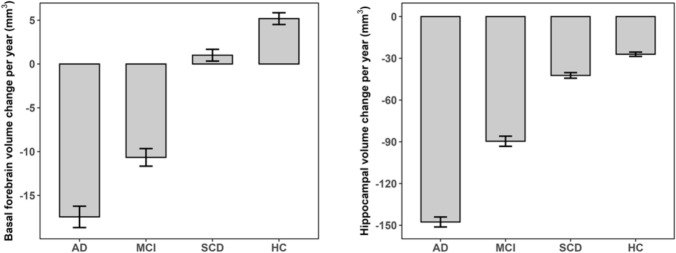
Rate of change in regional volumes across diagnostic groups*.* Error bars represent ± 1 standard error of the mean. (*N* = 223, AD = 19, MCI = 48, SCD = 74, HC = 82). *AD* Alzheimer’s disease, *HC* Healthy control, *MCI* Mild cognitive impairment, *SCD* Subjective cognitive decline

Figure 2 compares the annualized atrophy rates estimated in the mixed-effects models for the basal forebrain (left panel) and the hippocampus (right panel) across diagnostic groups. After adjusting for age and sex, ANCOVA models revealed significant group differences for both hippocampal (F(3, 1316) = 208.15, *p* < 0.001) and basal forebrain (F(3, 1316) = 71.81, *p* < 0.001) atrophy rates. Pairwise comparisons with Bonferroni correction for multiple testing showed that the AD group exhibited higher hippocampal atrophy than the MCI, SCD, and HC (p_Bonferroni-adjusted_ < 0.001). In the basal forebrain, AD patients also showed higher atrophy rates than other groups (p_Bonferroni-adjusted_ < 0.01; see Supplementary Tables 4 and 5).

### Longitudinal analysis

**Fig. 3:**
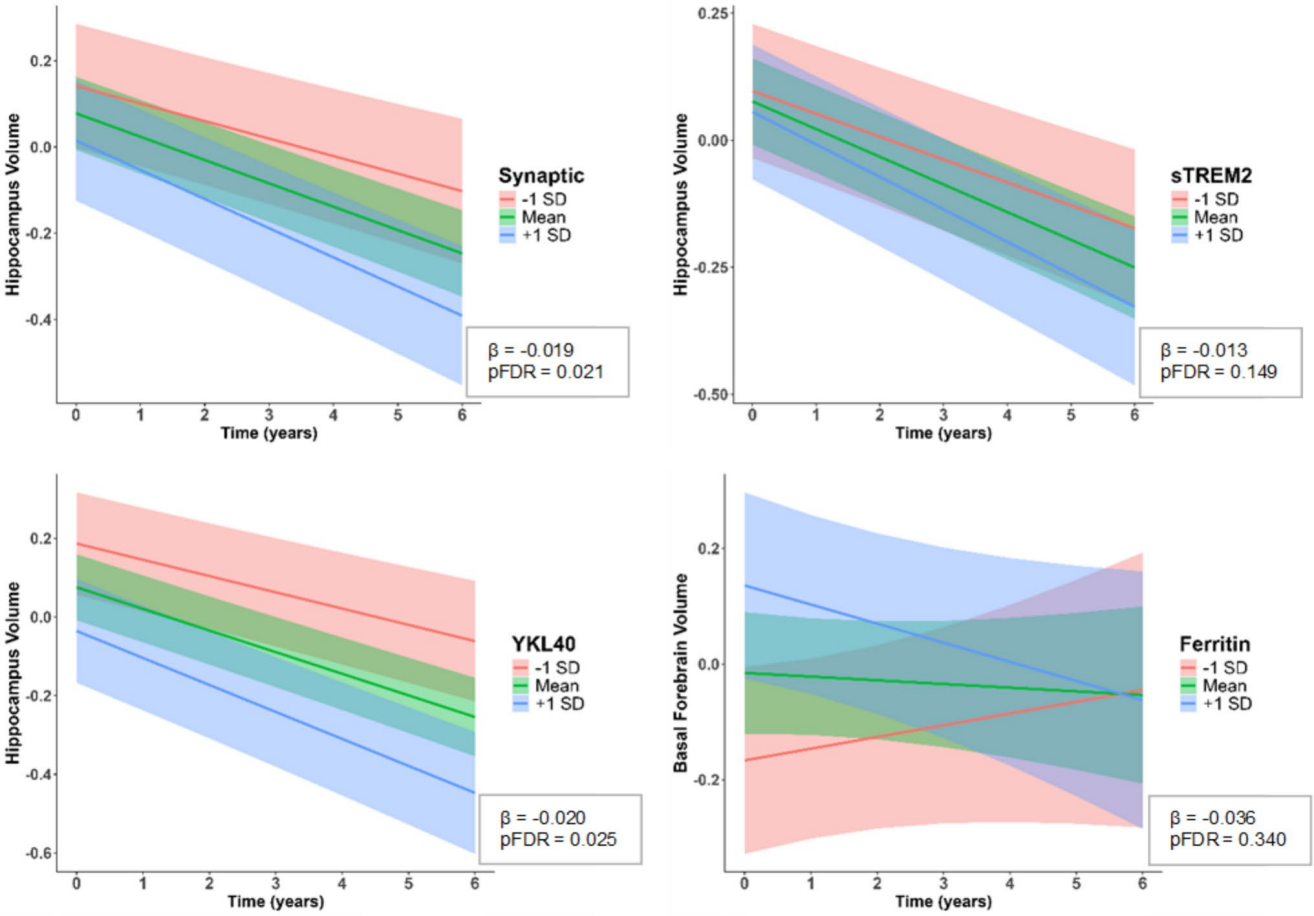
Longitudinal trajectories of hippocampal and basal forebrain volumes depending on biomarker levels*.* Predicted values of region-specific brain volumes were obtained from linear mixed-effects models including the interaction of biomarker with time while controlling for age, sex, education, and ApoE-ε4 status (*N* = 223, AD = 19, MCI = 48, SCD = 74, HC = 82). Random intercepts and random slopes for time were included to account for repeated measurements within individuals. The interaction terms were visualized using model-predicted trajectories stratified by biomarker levels at − 1 SD, mean, and + 1 SD, with 95% confidence intervals shown as shaded ribbons. *SD* standard deviation, *sTREM2* soluble Triggering Receptor Expressed on Myeloid cells 2

In the model predicting hippocampus volume, we observed negative time × biomarker interaction effects for the synaptic factor (*β* =  − 0.019, 95% CI [− 0.033, − 0.006], *p* = 0.005), sTREM2 (*β* =  − 0.013, 95% CI [− 0.025, − 0.001], *p* = 0.031), and YKL-40 (*β* =  − 0.020, 95% CI [− 0.034, − 0.007], *p* = 0.003) (Table 3, Fig. 3). When models with hippocampus were corrected for multiple comparisons, only synaptic factor and YKL-40 (pFDR = 0.021 and 0.025, respectively) remained significant. Participants with AD, MCI (both *p* < 0.001), and SCD (*p* < 0.05) showed greater decline in hippocampus volume compared to HC in the models with synaptic latent factor and YKL-40. Among A/T groups, the A + T + group showed a significant negative interaction with time (*β* =  − 0.068, 95% CI [− 0.103, − 0.033], *p* < 0.001 for synaptic and *β* =  − 0.072, 95% CI [− 0.106, − 0.039], *p* < 0.001 for YKL-40) relative to the A-T- group in both models. Age and sex were significant covariates for the synaptic factor, whereas sex, education, and ApoE-ε4 carrier status were significantly associated with hippocampal atrophy in the model with YKL-40. No significant interactions with time were observed for microglia, complement, chemokine, ferritin, or neurogranin (all *p* > 0.05) in the hippocampus models (see Supplementary Table 6). When the same model was repeated using the synaptic latent factor excluding ferritin, the interaction with time was not robust after correction (pFDR = 0.092; Supplementary Table 7).

**Table 3:** Results of linear mixed effects models for hippocampus and basal forebrain

	Hippocampus			Basal Forebrain
	Synaptic Latent Factor	sTREM2	YKL-40	Ferritin
*Intercept*				
Estimate	0.148	0.180	0.163	0.154
CI	[− 0.071, 0.368]	[− 0.037, 0.396]	[− 0.051, 0.378]	[− 0.101, 0.409]
t value	1.330	1.634	1.504	1.193
*Marker*				
Estimate	− 0.091	− 0.036	− 0.145*	0.103
CI	[− 0.213, 0.031]	[− 0.138, 0.066]	[− 0.257, −	[− 0.013, 0.219]
t value	− 1.465	− 0.697	0.034] − 2.567	1.754
*Time*				
Estimate	− 0.029*	− 0.021*	− 0.027*	0.055
CI	[− 0.051, − 0.007]	[− 0.042, − 0.00009]	[− 0.048, − 0.005]	[− 0.011, 0.121]
t value	− 2.589	− 1.982	− 2.468	1.642
*Marker:time*				
Estimate	− 0.019**	− 0.013*	− 0.020**	− 0.036*
CI	**[**− **0.033, **− **0.006]**	**[**− **0.025, **− **0.001]**	**[− 0.034, − 0.007]**	**[**− **0.072, **− **0.0002]**
t value	− **2.815**	− **2.161**	− **2.975**	− **1.984**
pFDR	**0.021**	**0.149**	**0.025**	**0.340**
*AD*				
Estimate	− 1.627***	− 1.679***	− 1.690***	− 1.020***
CI	[− 2.009, − 1.245]	[− 2.060, − 1.299]	[− 2.065, − 1.315]	[− 1.465, − 0.575]
t value	− 8.397	− 8.700	− 8.876	− 4.517
*MCI*				
Estimate	− 0.682***	− 0.694***	− 0.690***	− 0.578***
CI	[− 0.941, − 0.424]	[− 0.959, − 0.430]	[− 0.947, − 0.433]	[− 0.883, − 0.273]
t value	− 5.201	− 5.177	− 5.298	− 3.737
*SCD*				
Estimate	− 0.235*	− 0.238*	− 0.226*	− 0.075
CI	[− 0.451, − 0.018]	[− 0.459, − 0.017]	[− 0.440, −0.013]	[− 0.326, 0.176]
t value	− 2.136	− 2.118	− 2.087	− 0.589
*AD:time*				
Estimate	− 0.119***	− 0.131***	0.011	−0.012
CI	[− 0.170, − 0.067]	[− 0.182, − 0.080]	[− 0.079, 0.100]	[− 0.120, 0.096]
t value	− 4.523	− 5.072	0.232	− 0.221
*MCI:time*				
Estimate	− 0.068***	− 0.073***	− 0.316***	− 0.335***
CI	[− 0.101, − 0.035]	[− 0.106, − 0.039]	[− 0.414, − 0.218]	[− 0.449, − 0.221]
t value	− 4.038	− 4.247	− 6.375	− 5.806
*SCD:time*				
Estimate	− 0.022	− 0.025	− 0.078	− 0.143
CI	[− 0.047, 0.004]	[− 0.051, 0.001]	[− 0.281, 0.125]	[− 0.404, 0.119]
t value	− 1.679	− 1.924	− 0.756	− 1.075
*Sex(F)*				
Estimate	0.445***	0.459***	− 0.133***	− 0.104
CI	[0.254, 0.635]	[0.271, 0.647]	[− 0.184, − 0.083]	[− 0.270, 0.061]
t value	4.596	4.816	− 5.191	− 1.240
*Education*				
Estimate	0.014	0.012	− 0.068***	− 0.100
CI	[− 0.076, 0.105]	[− 0.079, 0.103]	[− 0.101, − 0.034]	[− 0.204, 0.004]
t value	0.311	0.263	− 4.014	− 1.897
*Age*				
Estimate	− 0.343***	− 0.346***	− 0.019	− 0.035
CI	[− 0.437, − 0.250]	[− 0.442, − 0.250]	[− 0.045, 0.006]	[− 0.114, 0.044]
t value	− 7.219	− 7.102	− 1.499	− 0.881
*ApoE4(+)*				
Estimate	− 0.038	− 0.066	0.434***	0.407***
CI	[− 0.259, 0.184]	[− 0.284, 0.151]	[0.246, 0.622]	[0.184, 0.631]
t value	− 0.335	− 0.600	4.560	3.590
*A/T(A + T-)*				
Estimate	− 0.063	− 0.054	− 0.109	− 0.231
CI	[− 0.329, 0.203]	[− 0.321, 0.213]	[− 0.376, 0.158]	[− 0.539, 0.077]
t value	− 0.466	− 0.400	− 0.805	− 1.477
*A/T(A-T +)*				
Estimate	− 0.044	− 0.111	− 0.028	− 0.231
CI	[− 0.347, 0.259]	[− 0.396, 0.175]	[− 0.318, 0.261]	[− 0.561, 0.099]
t value	− 0.287	− 0.763	− 0.193	− 1.382
*A/T(A + T +)*				
Estimate	− 0.247	− 0.300*	− 0.221	− 0.316*
CI	[− 0.524, 0.030]	[− 0.567, − 0.033]	[− 0.491, 0.050]	[− 0.625, − 0.006]
t value	− 1.758	− 2.218	− 1.608	− 2.010
*A/T(A + T-):time*				
Estimate	− 0.003	− 0.003	− 0.010	0.002
CI	[− 0.034, 0.028]	[− 0.035, 0.028]	[− 0.041, 0.021]	[− 0.093, 0.097]
t value	− 0.197	− 0.218	− 0.617	0.043
*A/T(A-T +):time*				
Estimate	0.010	− 0.003	0.005	− 0.024
CI	[− 0.028, 0.047]	[− 0.038, 0.033]	[− 0.031, 0.042]	[− 0.132, 0.084]
t value	0.504	− 0.146	0.292	− 0.439
*A/T(A + T +):time*				
Estimate	− 0.068***	− 0.078	− 0.072***	− 0.082
CI	[− 0.103, − 0.033]	[− 0.112, − 0.044]	[− 0.106, − 0.039]	[− 0.185, 0.022]
t value	− 3.808	− 4.575	− 4.240	− 1.560

Interaction effects of markers were highlighted in bold

Each column presents regression coefficients (estimate), confidence intervals (CI) and t values from a separate linear mixed-effects model, each involving either a latent factor or an individual biomarker.  *p < 0.05, ** *p* < 0.01, ***p < 0.001

*AD* Alzheimer’s disease, *A/T* Amyloid beta/Tau, *CI* Confidence interval, *HC* Healthy control; *MCI* Mild cognitive impairment, *SCD* Subjective cognitive decline

In the models predicting basal forebrain volume, negative time × biomarker interaction effects were found for ferritin (*β* = − 0.036, 95% CI [− 0.072, − 0.0002], *p* = 0.048) (Table 3). However, this interaction did not survive correction for multiple comparisons (pFDR = 0.340) among models predicting basal forebrain atrophy. No significant interactions were found for synaptic (both versions), microglia, complement, chemokine, sTREM2, YKL-40, or neurogranin in the basal forebrain models (all *p* > 0.05; see Supplementary Tables 7 and 8). Among other markers in the panel, only FABP3 in the hippocampus survived correction (*β* = − 0.020, 95% CI [− 0.034, − 0.007], pFDR = 0.025; see Supplementary Tables 9 and 10).

### Mediation analysis

In the mediation model with the synaptic latent factor, compared to the A-T- reference group, A + T- group showed lower synaptic factor levels (*β* = − 0.270, 95% CI [− 0.364, −0.177]), whereas both the A-T + (*β* = 0.867, 95% CI [0.750, 0.983]) and A + T + (*β* = 0.610, 95% CI [0.477, 0.736]) groups exhibited higher synaptic latent factor levels (Supplementary Table 11). Significant indirect effects were observed for all A/T groups. The A + T- group showed a small positive indirect effect on hippocampal atrophy through the synaptic latent marker (*β* = 0.040, 95% CI [0.023, 0.064]), whereas both the A-T + (*β* = − 0.128, 95% CI [− 0.183, − 0.083]) and A + T + (β = −0.090, 95% CI [−0.136, −0.054]) groups exhibited negative indirect effects. Direct effects (*β* = − 0.509, 95% CI [− 0.638, − 0.384]) and total effects (*β* = − 0.600, 95% CI [− 0.717, − 0.486]) were significant only for the A + T + group (*p* < 0.001, Table 4).

**Table 4:** Results of mediation effects for hippocampus

	Synaptic Latent Factor			YKL-40		
	Estimate	Std. (β)	95% CI	Estimate	Std. (β)	95% CI
*Indirect*						
A^+^T^−^	0.040***	0.015	[0.023, 0.064]	0.068***	0.026	[0.042, 0.101]
A^−^T^+^	− 0.128***	− 0.044	[− 0.183, − 0.083]	− 0.106***	− 0.037	[− 0.152, − 0.068]
A^+^T^+^	− 0.090***	− 0.041	[− 0.136, − 0.054]	− 0.100***	− 0.045	[− 0.145, − 0.064]
*Direct*						
A^+^T^−^	0.017	0.006	[− 0.095, 0.124]	− 0.011	− 0.004	[− 0.121, 0.102]
A^−^T^+^	0.027	0.009	[− 0.095, 0.144]	0.005	0.002	[− 0.110, 0.113]
A^+^T^+^	− 0.509***	− 0.229	[− 0.638, − 0.384]	− 0.500***	− 0.225	[− 0.628, − 0.382]
*Total*						
A^+^T^−^	0.057	0.022	[− 0.056, 0.169]	0.057	0.022	[− 0.056, 0.169]
A^−^T^+^	− 0.102	− 0.035	[− 0.210, − 0.004]	− 0.102	− 0.035	[− 0.210, − 0.004]
A^+^T^+^	− 0.600***	− 0.270	[− 0.717, − 0.486]	− 0.600***	− 0.270	[− 0.717, − 0.486]

Results of the mediation models of synaptic latent factor and YKL-40 for hippocampus. *p < 0.05, **p < 0.01, ***p < 0.001

*CI* Confidence interval, *Std. (β)* Standardized estimate (coefficient)

In the mediation model including YKL-40, all A/T groups showed significant differences relative to the A-T- reference group. The A + T- group exhibited lower YKL-40 levels (*β* = − 0.424, 95% CI [− 0.521, − 0.325]), whereas both the A-T + (*β* = 0.663, 95% CI [0.557, 0.767]) and A + T + (*β* = 0.623, 95% CI [0.465, 0.768]) groups showed elevated YKL-40. Significant indirect effects on hippocampal atrophy were observed for all A/T groups. The A + T- group showed a positive indirect effect (*β* = 0.068, 95% CI [0.042, 0.101]), while the A-T + group (*β* = − 0.106, 95% CI [− 0.152, − 0.068]) and the A + T + group (*β* = − 0.100, 95% CI [− 0.145, − 0.064]) exhibited negative indirect effects. The direct effect was significant only in the A + T + group (*β* = − 0.500, 95% CI [− 0.628, − 0.382]), with a total effect on hippocampal atrophy (*β* = − 0.600, 95% CI [− 0.717, − 0.486]).

## Discussion

In this study, we examined the relationships between CSF biomarkers and longitudinal atrophy in the hippocampus and basal forebrain. In our previous work, only the synaptic and microglial factors were associated with accelerated cognitive decline [28]. Here, we tested whether these latent constructs and individual biomarkers representing inflammation and synaptic dysfunction predict regional atrophy and mediate disease pathology in these regions. Our results showed that the synaptic latent factor and YKL-40 predicted longitudinal atrophy in the hippocampus and partially mediated A/T based disease pathology in this region.

The synaptic latent factor was composed of neurogranin, FABP3, and ferritin [28], and showed robust associations with hippocampal atrophy in both longitudinal and mediation models. When tested individually, neither ferritin nor neurogranin was significantly associated with hippocampal atrophy, whereas FABP3 showed a significant interaction effect. In contrast, the exploratory synaptic latent factor excluding ferritin did not survive multiple comparison correction in the longitudinal models.

Synaptic degeneration is one of the early  signs of hippocampal neurodegeneration in AD [16]. The significant indirect effects we observed in the hippocampus suggest that synaptic dysfunction contributes to neurodegeneration through pathways associated with amyloid and tau pathology. During disease progression, amyloid accumulation interferes with neurotransmitter release and receptor signaling, while tau impairs axonal transport and dendritic spines [50, 51]. These processes activate microglia and astrocytes, which may further contribute to synaptic vulnerability and reduced synaptic integrity [51–53]. As synapses deteriorate, synaptic proteins are released into the extracellular space, and their increased CSF concentrations reflect the extent of synaptic injury. In our cohort, the synaptic latent factor and neurogranin were elevated in the AD group compared to other diagnostic groups, consistent with prior reports showing increased CSF synaptic markers in the AD spectrum [17, 52]. Synaptic loss in the hippocampus occurs early in the disease and is closely linked to memory deficits [16, 52]. The regional vulnerability likely explains the robust association between the synaptic latent factor and hippocampal atrophy over time.

The mixed results of the two versions of the synaptic factor suggest a combination of biological and statistical influences. Including ferritin likely increased the shared variance among the biomarkers, reflecting overlapping aspects of synaptic degeneration and iron-mediated oxidative stress. This may have enhanced the sensitivity of the latent factor to hippocampal atrophy. Although ferritin reflects iron storage and metabolism rather than synaptic function, elevated ferritin and cortical iron levels have been linked to amyloid and tau pathology and clinical progression in AD [54–57]. In our cohort, ferritin levels were higher in the AD group, consistent with previous studies [31]. However, when examined individually, ferritin did not show a significant association with hippocampal atrophy. Together, these results suggest that the latent factor including ferritin captured broader neurodegenerative processes linked to synaptic vulnerability [58–62], which may explain why it showed the more robust association with atrophy in the hippocampus.

Among individual markers, YKL-40 showed the most consistent associations with hippocampal atrophy. It predicted structural decline and partially mediated the effect of the disease in this region. This aligns with research showing that astrocytic activation increases in temporal regions affected by tau pathology [63, 64]. As a marker of astrocytic activation, YKL-40 may represent a sustained response associated with disease pathology and neurodegeneration [20–22]. Our findings align with previous studies showing that elevated YKL-40 is associated with disease progression and cognitive decline across the AD continuum [64, 65]. Therefore, therapeutic approaches targeting astroglial neuroinflammation may help modify the trajectory of AD [66].

In contrast to the synaptic factor and YKL-40, the other markers were not significantly associated with longitudinal atrophy in either region. These findings align with research that inflammatory biomarkers often show heterogeneous and stage-dependent associations with neurodegeneration in AD [67]. For example, CSF sTREM2 can show dynamic changes across disease stages and differs depending on amyloid and tau pathology [68, 69]. Similarly, cytokine and chemokine markers, as well as components of the complement system, vary over the disease course [22, 70] and may not correspond directly to region-specific atrophy. Therefore, these biomarkers may not be very sensitive to neurodegenerative processes in regions such as the basal forebrain, as mixed findings in the literature have shown [26, 29]. Overall, the results reflect the temporal dynamics and biological heterogeneity of inflammatory and synaptic processes in AD, which show region-specific associations.

In our study, synaptic dysfunction and astroglial activation showed significant associations with A/T based pathology and hippocampal atrophy. The mediation effects we observed across A + T-, A-T +, and A + T+ groups suggest that these biomarkers are associated with hippocampal neurodegeneration at different stages of the disease. Although several associations were statistically significant, effect sizes were modest, consistent with the multifactorial nature of neurodegeneration. Overall, our results support the importance of synaptic dysfunction and astrocytic activation in AD and highlight the potential value of therapeutic strategies that simultaneously protect synapses and modulate amyloid, tau, and glial responses [71]. These pathways may also help identify individuals at higher risk for rapid temporal degeneration, although further validation in independent longitudinal cohorts is needed.

## Strengths and limitations

A major strength of our study is the use of latent factor modeling to understand directly unobservable neuroinflammatory domains in AD. This approach captured shared biological variance across biomarkers. We included a broad panel of CSF markers covering multiple inflammatory pathways. Combining this approach with longitudinal imaging data enabled us the assessment of how these markers relate to structural brain changes over time. The Bayesian CFA improved construct validity, and the longitudinal mixed-effects models provided robust estimates of atrophy trajectories. Another strength of our study is the longitudinal analysis of basal forebrain atrophy in relation to CSF neuroinflammatory markers. This area has been shown to be underexplored in systematic reviews. Finally, the mediation framework identified indirect pathways partially linking amyloid and tau pathology to neurodegeneration. Here, we interpreted the results cautiously and reported 95% confidence intervals to show estimate precision since the findings are associative rather than mechanistic.

However, several limitations should be acknowledged. First, all CSF biomarkers were measured only at baseline, limiting the ability to capture temporal changes in inflammatory markers. The latent factors were defined a priori, and this approach may have overlooked marker-specific effects. To address this, we additionally tested single biomarkers in separate models. Second, while the inclusion of ferritin increased the sensitivity of the synaptic factor, its role extends beyond synaptic biology and primarily reflects iron metabolism. Its contribution to the latent construct should be carefully interpreted. Another limitation is related to diagnostic grouping, which may not fully reflect biological disease stages. However, our models incorporated both clinical and biomarker-based grouping to integrate diagnosis with pathology. Finally, because participants were recruited from a specific research cohort and selective follow-up cannot be ruled out, our findings may not be fully generalizable to more heterogeneous, community-based populations. Therefore, replication in independent longitudinal cohorts is required.

## Conclusion

Our study showed that astroglial activation and synaptic dysfunction are associated with hippocampal atrophy. Mediation analyses further highlighted their roles in linking amyloid and tau pathology to neurodegeneration and suggest that these biomarkers may also act as partial intermediaries of structural decline. Future studies should replicate these results in larger and more diverse cohorts using longitudinal CSF assessments and multimodal imaging. Therapeutic approaches should consider targeting synaptic and glial pathways to modify disease progression.

## Supplementary Information

Below is the link to the electronic supplementary material.

Supplementary file1 (DOCX 215 KB)

## Data Availability

Data analyzed in this study are not publicly available.

## Abbreviations

Aβ: Amyloid-beta
AD: Alzheimer’s disease
CERAD: Consortium to Establish a Registry for Alzheimer’s disease
CSF: Cerebrospinal fluid
C4: Complement component 4
DZNE: German Center for Neurodegenerative Diseases
FABP-3: Fatty acid binding protein 3
HC: Healthy control
IL-6: Interleukin-6
IL-18: Interleukin-18
IP–10: Interferon gamma-induced protein 10
MCI: Mild cognitive impairment
MCP-1: Monocyte chemoattractant protein-1
MIF-1α: Macrophage migration inhibitory factor-1α
MMSE: Mini-Mental State Examination
MRI: Magnetic resonance imaging
pTau181: Phosphorylated tau 181
sAXL: Soluble AXL receptor tyrosine kinase
SCD: Subjective cognitive decline
sTREM2: Soluble triggering receptor expressed on myeloid cells 2
YKL-40: Chitinase-3-like protein 1
