# Supplementary material for: CSF biomarkers of neuroinflammation are associated with regional atrophy
Source: J Neurol. 2025 Dec 24;273(1):46. doi: 10.1007/s00415-025-13564-5 (PMC12738631; doi:10.1007/s00415-025-13564-5)
Supplement: Supplementary file 1 — Supplementary file1 (DOCX 215 KB) [file 415_2025_13564_MOESM1_ESM.docx]

**SUPPLEMENTARY MATERIAL**

**Title: “CSF Biomarkers of Neuroinflammation are Associated with Regional Atrophy”**

**Supplementary Table 1** Latent factors and single biomarkers

|  | **AD** | **MCI** | **SCD** | **HC** |
| --- | --- | --- | --- | --- |
| Synaptic (with ferritin) | 0.64 (0.53) | 0.09 (0.65) | -0.15 (0.54) | -0.13 (0.45) |
| Synaptic (excl. ferritin) | 1.20 (0.98) | 0.21 (1.17) | -0.19 (0.87) | -0.08 (0.78) |
| Microglia | 0.05 (0.48) | -0.02 (0.55) | -0.06 (0.59) | 0.08 (0.58) |
| Chemokine | 0.07 (0.55) | 0.05 (0.47) | 0.03 (0.40) | -0.07 (0.41) |
| Complement | 0.02 (0.56) | 0.07 (0.77) | 0.08 (0.96) | -0.15 (0.63) |
| sTREM2 (pg/ml) | 7165 (1706) | 6157 (1699) | 5881 (1275) | 6319 (1683) |
| Neurogranin (pg/ml) | 656 (206) | 467 (236) | 395 (157) | 434 (154) |
| YKL40 (ng/ml) | 421 (124) | 391 (158) | 350 (136) | 337 (118) |
| Ferritin (ng/ml) | 38 (14) | 28 (10) | 28 (10) | 27 (9) |
| Hippocampus volume | 4516 (799) | 5674 (949) | 6330 (746) | 6572 (720) |
| Basal forebrain volume | 2172 (243) | 2371 (321) | 2576 (298) | 2570 (273) |
| MRI follow-up (in years) | 1.092 [0.866 - 4.767] | 1.093 [0.863 - 4.260] | 1.862 [0.614 - 4.978] | 1.956 [0.671 - 4.858] |

Baseline data is presented as mean (SD) for biomarkers (N = 227). MRI follow-up data in years were presented as median [min - max].


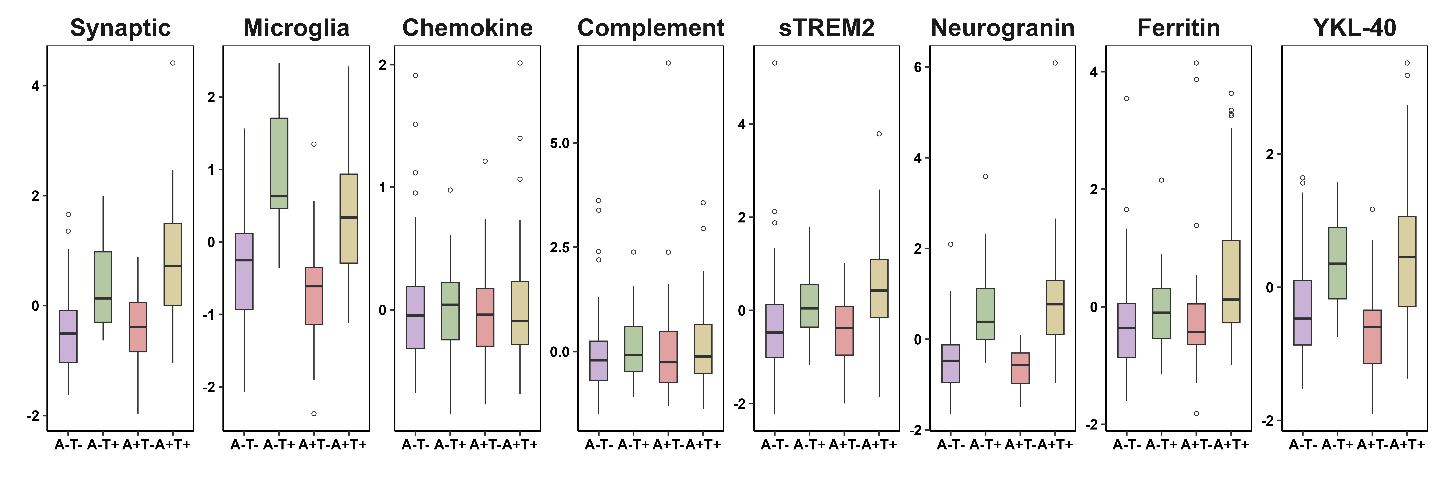


**Supplementary Figure 1** Levels of markers across A/T groups

**Supplementary Table 2** ANCOVA results of markers across diagnostic groups

| **Marker** | **Term** | **df** | **Sum Sq** | **Mean Sq** | **F** | **p** |
| --- | --- | --- | --- | --- | --- | --- |
| Chemokine | Diagnosis | 3 | 0.823 | 0.274 | 1.453 | 0.228 |
| Chemokine | Age | 1 | 0.798 | 0.798 | 4.223 | **0.041** |
| Chemokine | Sex | 1 | 0.016 | 0.016 | 0.083 | 0.773 |
| Chemokine | Residuals | 218 | 41.179 | 0.189 |  |  |
| Complement | Diagnosis | 3 | 2.796 | 0.932 | 1.572 | 0.197 |
| Complement | Age | 1 | 0.184 | 0.184 | 0.311 | 0.578 |
| Complement | Sex | 1 | 4.584 | 4.584 | 7.733 | **0.006** |
| Complement | Residuals | 218 | 129.218 | 0.593 |  |  |
| Ferritin | Diagnosis | 3 | 1851.221 | 617.074 | 5.886 | **<0.001** |
| Ferritin | Age | 1 | 1434.15 | 1434.15 | 13.679 | **<0.001** |
| Ferritin | Sex | 1 | 97.187 | 97.187 | 0.927 | 0.337 |
| Ferritin | Residuals | 218 | 22855.57 | 104.842 |  |  |
| Microglia | Diagnosis | 3 | 0.837 | 0.279 | 0.876 | 0.454 |
| Microglia | Age | 1 | 1.867 | 1.867 | 5.865 | **0.016** |
| Microglia | Sex | 1 | 0.252 | 0.252 | 0.792 | 0.375 |
| Microglia | Residuals | 218 | 69.393 | 0.318 |  |  |
| Neurogranin | Diagnosis | 3 | 1069269 | 356423.1 | 10.795 | **<0.001** |
| Neurogranin | Age | 1 | 7762.115 | 7762.115 | 0.235 | 0.628 |
| Neurogranin | Sex | 1 | 16998.49 | 16998.49 | 0.515 | 0.474 |
| Neurogranin | Residuals | 218 | 7197483 | 33015.98 |  |  |
| Synaptic | Diagnosis | 3 | 11.205 | 3.735 | 13.383 | **<0.001** |
| Synaptic | Age | 1 | 2.144 | 2.144 | 7.683 | **0.006** |
| Synaptic | Sex | 1 | 1.215 | 1.215 | 4.355 | **0.038** |
| Synaptic | Residuals | 218 | 60.842 | 0.279 |  |  |
| YKL40 | Diagnosis | 3 | 160683.6 | 53561.19 | 3.325 | **0.021** |
| YKL40 | Age | 1 | 504289 | 504289 | 31.303 | **<0.001** |
| YKL40 | Sex | 1 | 23565.54 | 23565.54 | 1.463 | 0.228 |
| YKL40 | Residuals | 218 | 3511930 | 16109.77 |  |  |
| sTREM2 | Diagnosis | 3 | 28797615 | 9599205 | 4.119 | **0.007** |
| sTREM2 | Age | 1 | 37271028 | 37271028 | 15.991 | **<0.001** |
| sTREM2 | Sex | 1 | 90963.43 | 90963.43 | 0.039 | 0.844 |
| sTREM2 | Residuals | 218 | 5080000 | 2330687 |  |  |

**Supplementary Table 3** Pairwise comparisons of markers across groups

| **Marker** | **Contrast** | **Estimate** | **Standard Error** | **df** | **Lower CL** | **Upper CL** | **t ratio** | **P** | **P_Bonferroni corrected_** |
| --- | --- | --- | --- | --- | --- | --- | --- | --- | --- |
| **Chemokine** | HC - AD | -0.091 | 0.114 | 218 | -0.316 | 0.134 | -0.797 | 0.427 | 1.000 |
| **Chemokine** | HC - MCI | -0.089 | 0.083 | 218 | -0.254 | 0.075 | -1.072 | 0.285 | 1.000 |
| **Chemokine** | HC - SCD | -0.068 | 0.072 | 218 | -0.209 | 0.073 | -0.945 | 0.346 | 1.000 |
| **Chemokine** | MCI - AD | -0.002 | 0.119 | 218 | -0.236 | 0.232 | -0.013 | 0.990 | 1.000 |
| **Chemokine** | SCD - AD | -0.023 | 0.113 | 218 | -0.245 | 0.199 | -0.208 | 0.836 | 1.000 |
| **Chemokine** | SCD - MCI | -0.022 | 0.081 | 218 | -0.181 | 0.138 | -0.27 | 0.787 | 1.000 |
| **Complement** | HC - AD | -0.135 | 0.202 | 218 | -0.534 | 0.264 | -0.666 | 0.506 | 1.000 |
| **Complement** | HC - MCI | -0.15 | 0.148 | 218 | -0.442 | 0.141 | -1.015 | 0.311 | 1.000 |
| **Complement** | HC - SCD | -0.175 | 0.127 | 218 | -0.425 | 0.075 | -1.38 | 0.169 | 1.000 |
| **Complement** | MCI - AD | 0.015 | 0.21 | 218 | -0.399 | 0.43 | 0.073 | 0.942 | 1.000 |
| **Complement** | SCD - AD | 0.04 | 0.2 | 218 | -0.353 | 0.434 | 0.202 | 0.840 | 1.000 |
| **Complement** | SCD - MCI | 0.025 | 0.143 | 218 | -0.258 | 0.307 | 0.174 | 0.862 | 1.000 |
| **Ferritin** | HC - AD | -8.21 | 2.692 | 218 | -13.516 | -2.904 | -3.05 | 0.003 | **0.015** |
| **Ferritin** | HC - MCI | 1.525 | 1.967 | 218 | -2.352 | 5.402 | 0.775 | 0.439 | 1.000 |
| **Ferritin** | HC - SCD | 0.648 | 1.687 | 218 | -2.676 | 3.972 | 0.384 | 0.701 | 1.000 |
| **Ferritin** | MCI - AD | -9.735 | 2.797 | 218 | -15.248 | -4.222 | -3.48 | <0.001 | **0.004** |
| **Ferritin** | SCD - AD | -8.858 | 2.654 | 218 | -14.089 | -3.628 | -3.338 | <0.001 | **0.006** |
| **Ferritin** | SCD - MCI | 0.877 | 1.907 | 218 | -2.881 | 4.635 | 0.46 | 0.646 | 1.000 |
| **Microglia** | HC - AD | 0.143 | 0.148 | 218 | -0.15 | 0.435 | 0.962 | 0.337 | 1.000 |
| **Microglia** | HC - MCI | 0.187 | 0.108 | 218 | -0.027 | 0.4 | 1.721 | 0.087 | 0.520 |
| **Microglia** | HC - SCD | 0.2 | 0.093 | 218 | 0.017 | 0.383 | 2.151 | 0.033 | 0.195 |
| **Microglia** | MCI - AD | -0.044 | 0.154 | 218 | -0.348 | 0.26 | -0.285 | 0.776 | 1.000 |
| **Microglia** | SCD - AD | -0.057 | 0.146 | 218 | -0.345 | 0.231 | -0.391 | 0.696 | 1.000 |
| **Microglia** | SCD - MCI | -0.013 | 0.105 | 218 | -0.22 | 0.194 | -0.127 | 0.899 | 1.000 |
| **Neurogranin** | HC - AD | -216.052 | 47.775 | 218 | -310.211 | -121.892 | -4.522 | <0.001 | **<0.001** |
| **Neurogranin** | HC - MCI | -18.363 | 34.908 | 218 | -87.164 | 50.438 | -0.526 | 0.599 | 1.000 |
| **Neurogranin** | HC - SCD | 46.123 | 29.932 | 218 | -12.87 | 105.116 | 1.541 | 0.125 | 0.749 |
| **Neurogranin** | MCI - AD | -197.688 | 49.639 | 218 | -295.522 | -99.855 | -3.983 | <0.001 | **<0.001** |
| **Neurogranin** | SCD - AD | -262.175 | 47.096 | 218 | -354.997 | -169.352 | -5.567 | <0.001 | **<0.001** |
| **Neurogranin** | SCD - MCI | -64.486 | 33.834 | 218 | -131.169 | 2.197 | -1.906 | 0.058 | 0.348 |
| **Synaptic** | HC - AD | -0.675 | 0.139 | 218 | -0.948 | -0.401 | -4.857 | <0.001 | **<0.001** |
| **Synaptic** | HC - MCI | -0.106 | 0.101 | 218 | -0.306 | 0.094 | -1.048 | 0.296 | 1.000 |
| **Synaptic** | HC - SCD | 0.092 | 0.087 | 218 | -0.079 | 0.264 | 1.06 | 0.290 | 1.000 |
| **Synaptic** | MCI - AD | -0.568 | 0.144 | 218 | -0.853 | -0.284 | -3.938 | <0.001 | **<0.001** |
| **Synaptic** | SCD - AD | -0.767 | 0.137 | 218 | -1.037 | -0.497 | -5.601 | <0.001 | **<0.001** |
| **Synaptic** | SCD - MCI | -0.199 | 0.098 | 218 | -0.392 | -0.005 | -2.019 | 0.045 | 0.268 |
| **YKL40** | HC - AD | -35.855 | 33.372 | 218 | -101.628 | 29.918 | -1.074 | 0.284 | 1.000 |
| **YKL40** | HC - MCI | -10.524 | 24.384 | 218 | -58.584 | 37.535 | -0.432 | 0.666 | 1.000 |
| **YKL40** | HC - SCD | 14.406 | 20.908 | 218 | -26.802 | 55.614 | 0.689 | 0.492 | 1.000 |
| **YKL40** | MCI - AD | -25.33 | 34.674 | 218 | -93.67 | 43.009 | -0.731 | 0.466 | 1.000 |
| **YKL40** | SCD - AD | -50.261 | 32.898 | 218 | -115.1 | 14.578 | -1.528 | 0.128 | 0.768 |
| **YKL40** | SCD - MCI | -24.93 | 23.634 | 218 | -71.51 | 21.649 | -1.055 | 0.293 | 1.000 |
| **sTREM2** | HC - AD | -503.563 | 401.402 | 218 | -1294.69 | 287.562 | -1.255 | 0.211 | 1.000 |
| **sTREM2** | HC - MCI | 496.33 | 293.298 | 218 | -81.732 | 1074.393 | 1.692 | 0.092 | 0.552 |
| **sTREM2** | HC - SCD | 657.373 | 251.486 | 218 | 161.717 | 1153.029 | 2.614 | 0.010 | 0.057 |
| **sTREM2** | MCI - AD | -999.894 | 417.063 | 218 | -1821.89 | -177.901 | -2.397 | 0.017 | 0.104 |
| **sTREM2** | SCD - AD | -1160.94 | 395.701 | 218 | -1940.83 | -381.047 | -2.934 | 0.004 | **0.022** |
| **sTREM2** | SCD - MCI | -161.043 | 284.27 | 218 | -721.311 | 399.226 | -0.567 | 0.572 | 1.000 |

**Supplementary Table 4** ANCOVA results of rate of change in regions across groups

| **Outcome** | **Term** | **df** | **Sum sq** | **Mean sq** | **F value** | **P value** |
| --- | --- | --- | --- | --- | --- | --- |
| **Hippocampus** | **Diagnosis** | 3 | 980871.6 | 326957.2 | 208.158 | **< 0.001** |
| **Hippocampus** | **age** | 1 | 418248.7 | 418248.7 | 266.279 | **< 0.001** |
| **Hippocampus** | **sex** | 1 | 10499.3 | 10499.3 | 6.684 | **0.009** |
| **Hippocampus** | **Residuals** | 1316 | 2067066.4 | 1570.7 |  |  |
| **Basal Forebrain** | **Diagnosis** | 3 | 45183.7 | 15061.2 | 71.819 | **< 0.001** |
| **Basal Forebrain** | **age** | 1 | 20167.1 | 20167.1 | 96.166 | **< 0.001** |
| **Basal Forebrain** | **sex** | 1 | 255.1 | 255.1 | 1.217 | 0.27 |
| **Basal Forebrain** | **Residuals** | 1316 | 275979 | 209.7 |  |  |

**Supplementary Table 5** Pairwise comparisons of rate of change in basal forebrain and hippocampus

| **Region** | **Contrast** | **Estimate** | **SE** | **df** | **T ratio** | **P** | **P_Bonferroni corrected_** |
| --- | --- | --- | --- | --- | --- | --- | --- |
| **Hippocampus** | **SCD - HC** | -6.20948489 | 2.59172351 | 1316 | -2.39589018 | 0.017 | 0.1 |
| **Hippocampus** | **MCI - HC** | -48.0408235 | 3.14030062 | 1316 | -15.2981607 | < .001 | **< .001** |
| **Hippocampus** | **MCI - SCD** | -41.8313386 | 3.07808562 | 1316 | -13.5900504 | < .001 | **< .001** |
| **Hippocampus** | **AD - HC** | -104.738837 | 4.9640782 | 1316 | -21.0993528 | < .001 | **< .001** |
| **Hippocampus** | **AD - SCD** | -98.5293525 | 4.92391068 | 1316 | -20.0103858 | < .001 | **< .001** |
| **Hippocampus** | **AD - MCI** | -56.6980139 | 5.18133315 | 1316 | -10.9427463 | < .001 | **< .001** |
| **Basal Forebrain** | **SCD - HC** | -2.37808244 | 0.94699885 | 1316 | -2.51117774 | 0.012 | 0.073 |
| **Basal Forebrain** | **MCI - HC** | -13.167677 | 1.14744535 | 1316 | -11.4756463 | < .001 | **< .001** |
| **Basal Forebrain** | **MCI - SCD** | -10.7895946 | 1.12471239 | 1316 | -9.59320326 | < .001 | **< .001** |
| **Basal Forebrain** | **AD - HC** | -19.2083103 | 1.81384177 | 1316 | -10.5898489 | < .001 | **< .001** |
| **Basal Forebrain** | **AD - SCD** | -16.8302279 | 1.79916482 | 1316 | -9.35446696 | < .001 | **< .001** |
| **Basal Forebrain** | **AD - MCI** | -6.04063329 | 1.89322531 | 1316 | -3.19065737 | 0.001 | **0.009** |

**Supplementary Table 6** Results of Mixed Effect Models for Hippocampus

| **Hippocampus** | | | | | | |
| --- | --- | --- | --- | --- | --- | --- |
|  | | **Microglia** | **Chemokine** | **Complement** | **Neurogranin** | **Ferritin** |
| **Intercept** | Estimate  CI  t value | 0.188  [-0.028, 0.403]  1.719 | 0.184  [-0.033, 0.400]  1.673 | 0.181  [-0.035, 0.396]  1.655 | 0.191  [-0.031, 0.412]  1.697 | 0.182  [-0.036, 0.400]  1.649 |
| **Marker** | Estimate  CI  t value | 0.088  [-0.024, 0.201]  1.545 | -0.049  [-0.253, 0.155]  -0.477 | -0.074  [-0.164, 0.015]  -1.634 | 0.010  [-0.107, 0.127]  0.173 | -0.007  [-0.107, 0.092]  -0.142 |
| **Time** | Estimate  CI  t value | -0.019  [-0.040, 0.003]  -1.732 | -0.020  [-0.041, 0.002]  -1.806 | -0.021  [-0.042, 0.001]  -1.887 | -0.022*  [-0.044, -0.001]  -2.028 | -0.022*  [-0.043, -0.000]  -1.983 |
| **Marker:time** | **Estimate**  **CI**  **t value**  **p value**  **pFDR** | **0.000**  **[-0.014, 0.014]**  **0.029**  **0.976**  **0.976** | **-0.008**  **[-0.035, 0.019]**  **-0.572**  **0.567**  **0.757** | **-0.005**  **[-0.016, 0.006]**  **-0.929**  **0.354**  **0.708** | **-0.010**  **[-0.023, 0.004]**  **-1.372**  **0.171**  **0.417** | **-0.008**  **[-0.019, 0.004]**  **-1.343**  **0.180**  **0.417** |
| **AD** | Estimate  CI  t value | -1.619***  [-2.004, -1.234]  -8.294 | -1.674***  [-2.054, -1.293]  -8.671 | -1.674***  [-2.051, -1.296]  -8.735 | -1.680***  [-2.062, -1.297]  -8.656 | -1.671***  [-2.051, -1.290]  -8.658 |
| **MCI** | Estimate  CI  t value | -0.635***  [-0.900, -0.371]  -4.739 | -0.676***  [-0.936, -0.415]  -5.116 | -0.668***  [-0.927, -0.410]  -5.097 | -0.679***  [-0.939, -0.418]  -5.130 | -0.680***  [-0.942, -0.419]  -5.128 |
| **SCD** | Estimate  CI  t value | -0.202  [-0.420, 0.015]  -1.833 | -0.224*  [-0.440, -0.007]  -2.031 | -0.212  [-0.428, 0.004]  -1.932 | -0.224*  [-0.442, -0.006]  -2.024 | -0.224*  [-0.441, -0.007]  -2.037 |
| **AD:time** | Estimate  CI  t value | -0.134***  [-0.186, -0.082]  -5.085 | -0.133***  [-0.185, -0.081]  -5.064 | -0.134***  [-0.186, -0.083]  -5.140 | -0.128***  [-0.180, -0.075]  -4.818 | -0.127***  [-0.180, -0.075]  -4.803 |
| **MCI:time** | Estimate  CI  t value | -0.069***  [-0.103, -0.034]  -3.952 | -0.068***  [-0.102, -0.034]  -3.930 | -0.068***  [-0.102, -0.034]  -3.945 | -0.070***  [-0.104, -0.036]  -4.073 | -0.070***  [-0.104, -0.036]  -4.063 |
| **SCD:time** | Estimate  CI  t value | -0.022  [-0.048, 0.004]  -1.675 | -0.021  [-0.048, 0.005]  -1.607 | -0.021  [-0.047, 0.006]  -1.560 | -0.024  [-0.050, 0.002]  -1.825 | -0.021  [-0.047, 0.005]  -1.591 |
| **Sex(F)** | Estimate  CI  t value | 0.476***  [0.288, 0.663]  4.989 | 0.458***  [0.270, 0.645]  4.804 | 0.433***  [0.243, 0.624]  4.495 | 0.463***  [0.274, 0.651]  4.843 | 0.460***  [0.272, 0.648]  4.821 |
| **Education** | Estimate  CI  t value | 0.006  [-0.084, 0.096]  0.141 | 0.011  [-0.079, 0.101]  0.241 | 0.008  [-0.082, 0.098]  0.168 | 0.008  [-0.083, 0.099]  0.173 | 0.010  [-0.081, 0.101]  0.223 |
| **Age** | Estimate  CI  t value | -0.356***  [-0.449, -0.263]  -7.527 | -0.344***  [-0.438, -0.250]  -7.211 | -0.345***  [-0.438, -0.253]  -7.340 | -0.345***  [-0.438, -0.251]  -7.278 | -0.349***  [-0.444, -0.254]  -7.206 |
| **ApoE4(+)** | Estimate  CI  t value | -0.090  [-0.295, 0.115]  -0.862 | -0.071  [-0.277, 0.135]  -0.677 | -0.064  [-0.269, 0.141]  -0.616 | -0.090  [-0.304, 0.125]  -0.825 | -0.081  [-0.300, 0.139]  -0.725 |
| **ATA+T-** | Estimate  CI  t value | -0.011  [-0.280, 0.258]  -0.079 | -0.053  [-0.320, 0.213]  -0.396 | -0.040  [-0.305, 0.224]  -0.300 | -0.045  [-0.313, 0.223]  -0.332 | -0.047  [-0.313, 0.219]  -0.347 |
| **ATA-T+** | Estimate  CI  t value | -0.239  [-0.555, 0.077]  -1.493 | -0.125  [-0.409, 0.158]  -0.872 | -0.104  [-0.387, 0.179]  -0.727 | -0.140  [-0.452, 0.172]  -0.883 | -0.124  [-0.409, 0.160]  -0.861 |
| **ATA+T+** | Estimate  CI  t value | -0.399**  [-0.675, -0.123]  -2.851 | -0.331*  [-0.594, -0.069]  -2.490 | -0.319*  [-0.580, -0.058]  -2.412 | -0.337*  [-0.625, -0.048]  -2.303 | -0.319*  [-0.584, -0.053]  -2.366 |
| **ATA+T-:time** | Estimate  CI  t value | -0.003  [-0.035, 0.029]  -0.184 | -0.003  [-0.035, 0.028]  -0.203 | -0.001  [-0.033, 0.031]  -0.079 | -0.005  [-0.036, 0.027]  -0.300 | -0.002  [-0.033, 0.030]  -0.124 |
| **ATA-T+:time** | Estimate  CI  t value | -0.010  [-0.050, 0.031]  -0.467 | -0.009  [-0.045, 0.027]  -0.481 | -0.007  [-0.043, 0.029]  -0.382 | 0.001  [-0.038, 0.039]  0.034 | -0.006  [-0.042, 0.029]  -0.356 |
| **ATA+T+:time** | Estimate  CI  t value | -0.090***  [-0.124, -0.055]  -5.095 | -0.090***  [-0.122, -0.057]  -5.473 | -0.088***  [-0.120, -0.055]  -5.328 | -0.079***  [-0.115, -0.043]  -4.344 | -0.083***  [-0.116, -0.049]  -4.850 |

*p<0.05, ** p<0.01, ***p<0.001

**Supplementary Table 7** Results of Mixed Effect Models for Synaptic Latent Factor without Ferritin

| **Synaptic Latent Factor without Ferritin** | | | |
| --- | --- | --- | --- |
|  |  | **Hippocampus** | **Basal Forebrain** |
| **Intercept** | Estimate  CI  t value | 0.166  [-0.054, 0.386]  1.487 | 0.141  [-0.118, 0.401]  1.077 |
| **Marker** | Estimate  CI  t value | -0.048  [-0.164, 0.069]  -0.805 | 0.040  [-0.097, 0.177]  0.574 |
| **Time** | Estimate  CI  t value | -0.026*  [-0.047, -0.004]  -2.327 | 0.066  [-0.002, 0.134]  1.910 |
| **Marker:time** | **Estimate**  **CI**  **t value**  **p value**  **pFDR** | **-0.016***  **[-0.029, -0.002]**  **-2.293**  **0.0230***  **0.092** | **-0.005**  **[-0.046, 0.036]**  **-0.242**  **0.809**  **0.809** |
| **AD** | Estimate  CI  t value | -1.650***  [-2.033, -1.267]  -8.491 | -1.032***  [-1.483, -0.581]  -4.510 |
| **MCI** | Estimate  CI  t value | -0.684***  [-0.944, -0.425]  -5.194 | -0.608***  [-0.913, -0.304]  -3.934 |
| **SCD** | Estimate  CI  t value | -0.233*  [-0.450, -0.015]  -2.108 | -0.083  [-0.336, 0.171]  -0.642 |
| **AD:time** | Estimate  CI  t value | -0.122***  [-0.174, -0.070]  -4.618 | -0.134  [-0.301, 0.033]  -1.577 |
| **MCI:time** | Estimate  CI  t value | -0.069***  [-0.103, -0.036]  -4.074 | -0.097  [-0.202, 0.009]  -1.811 |
| **SCD:time** | Estimate  CI  t value | -0.023  [-0.049, 0.002]  -1.792 | -0.042  [-0.122, 0.037]  -1.048 |
| **Sex(F)** | Estimate  CI  t value | 0.455***  [0.266, 0.645]  4.730 | 0.409***  [0.182, 0.637]  3.549 |
| **Education** | Estimate  CI  t value | 0.012  [-0.079, 0.103]  0.260 | -0.003  [-0.112, 0.106]  -0.053 |
| **Age** | Estimate  CI  t value | -0.347***  [-0.440, -0.254]  -7.337 | -0.314***  [-0.425, -0.202]  -5.523 |
| **ApoE4(+)** | Estimate  CI  t value | -0.063  [-0.282, 0.157]  -0.564 | -0.082  [-0.345, 0.181]  -0.613 |
| **ATA+T-** | Estimate  CI  t value | -0.057  [-0.325, 0.210]  -0.422 | -0.232  [-0.545, 0.080]  -1.466 |
| **ATA-T+** | Estimate  CI  t value | -0.077  [-0.386, 0.233]  -0.488 | -0.246  [-0.608, 0.116]  -1.341 |
| **ATA+T+** | Estimate  CI  t value | -0.276  [-0.559, 0.008]  -1.918 | -0.314  [-0.646, 0.019]  -1.860 |
| **ATA+T-:time** | Estimate  CI  t value | -0.004  [-0.036, 0.027]  -0.279 | -0.003  [-0.099, 0.093]  -0.058 |
| **ATA-T+:time** | Estimate  CI  t value | 0.007  [-0.031, 0.046]  0.385 | -0.030  [-0.147, 0.087]  -0.506 |
| **ATA+T+:time** | Estimate  CI  t value | -0.071***  [-0.107, -0.035]  -3.920 | -0.106  [-0.218, 0.006]  -1.864 |

*p<0.05, ** p<0.01, ***p<0.001

**Supplementary Table 8** Results of Mixed Effect Models for Basal forebrain

| **Basal Forebrain** | | | | | | | | |
| --- | --- | --- | --- | --- | --- | --- | --- | --- |
|  | | **Synaptic** | **sTREM2** | **Microglia** | **Chemokine** | **Complement** | **Neurogranin** | **YKL40** |
| **Intercept** | Estimate  CI  t value | 0.131  [-0.128, 0.391]  0.997 | 0.131  [-0.124, 0.386]  1.010 | 0.130  [-0.123, 0.383]  1.012 | 0.125  [-0.131, 0.380]  0.963 | 0.124  [-0.130, 0.379]  0.963 | 0.159  [-0.101, 0.418]  1.204 | 0.120  [-0.135, 0.374]  0.925 |
| **Marker** | Estimate  CI  t value | 0.012  [-0.132, 0.156]  0.166 | 0.029  [-0.091, 0.148]  0.469 | 0.108  [-0.024, 0.239]  1.614 | -0.009  [-0.249, 0.231]  -0.074 | -0.021  [-0.127, 0.084]  -0.397 | 0.078  [-0.058, 0.214]  1.127 | -0.084  [-0.216, 0.049]  -1.247 |
| **Time** | Estimate  CI  t value | 0.060  [-0.009, 0.129]  1.716 | 0.065  [-0.001, 0.131]  1.957 | 0.066*  [0.000, 0.132]  1.987 | 0.060  [-0.005, 0.125]  1.821 | 0.059  [-0.008, 0.125]  1.750 | 0.071*  [0.003, 0.138]  2.073 | 0.054  [-0.012, 0.121]  1.606 |
| **Marker:time** | **Estimate**  **CI**  **t value**  **p value**  **pFDR** | **-0.016**  **[-0.058, 0.027]**  **-0.715**  **0.475**  **0.634** | **-0.015**  **[-0.051, 0.022]**  **-0.797**  **0.426**  **0.553** | **-0.008**  **[-0.050, 0.034]**  **-0.373**  **0.709**  **0.709** | **-0.075**  **[-0.159, 0.008]**  **-1.782**  **0.076**  **0.305** | **-0.023**  **[-0.056, 0.011]**  **-1.326**  **0.186**  **0.373** | **0.007**  **[-0.035, 0.049]**  **0.332**  **0.740**  **0.740** | **-0.034**  **[-0.076, 0.008]**  **-1.605**  **0.110**  **0.430** |
| **AD** | Estimate  CI  t value | -1.018***  [-1.470, -0.566]  -4.440 | -1.002***  [-1.450, -0.554]  -4.410 | -0.942***  [-1.395, -0.489]  -4.100 | -1.010***  [-1.458, -0.562]  -4.443 | -1.015***  [-1.461, -0.568]  -4.479 | -1.047***  [-1.495, -0.598]  -4.598 | -1.023***  [-1.470, -0.576]  -4.513 |
| **MCI** | Estimate  CI  t value | -0.613***  [-0.918, -0.308]  -3.964 | -0.598***  [-0.908, -0.288]  -3.799 | -0.558***  [-0.868, -0.248]  -3.548 | -0.613***  [-0.919, -0.308]  -3.954 | -0.612***  [-0.917, -0.307]  -3.960 | -0.599***  [-0.903, -0.294]  -3.873 | -0.624***  [-0.929, -0.320]  -4.040 |
| **SCD** | Estimate  CI  t value | -0.088  [-0.341, 0.166]  -0.683 | -0.077  [-0.335, 0.181]  -0.587 | -0.061  [-0.314, 0.193]  -0.472 | -0.088  [-0.342, 0.165]  -0.687 | -0.085  [-0.338, 0.168]  -0.664 | -0.073  [-0.326, 0.180]  -0.568 | -0.093  [-0.345, 0.160]  -0.725 |
| **AD:time** | Estimate  CI  t value | -0.125  [-0.292, 0.042]  -1.475 | -0.133  [-0.297, 0.030]  -1.606 | -0.142  [-0.306, 0.022]  -1.702 | -0.125  [-0.288, 0.037]  -1.519 | -0.137  [-0.300, 0.025]  -1.665 | -0.144  [-0.311, 0.023]  -1.704 | -0.134  [-0.296, 0.029]  -1.622 |
| **MCI:time** | Estimate  CI  t value | -0.096  [-0.201, 0.009]  -1.798 | -0.101  [-0.207, 0.005]  -1.882 | -0.099  [-0.205, 0.007]  -1.852 | -0.088  [-0.192, 0.017]  -1.647 | -0.092  [-0.197, 0.013]  -1.736 | -0.097  [-0.202, 0.009]  -1.808 | -0.095  [-0.200, 0.010]  -1.791 |
| **SCD:time** | Estimate  CI  t value | -0.041  [-0.120, 0.038]  -1.021 | -0.045  [-0.124, 0.035]  -1.111 | -0.043  [-0.123, 0.036]  -1.072 | -0.032  [-0.112, 0.047]  -0.807 | -0.034  [-0.114, 0.046]  -0.841 | -0.041  [-0.120, 0.039]  -1.005 | -0.037  [-0.116, 0.042]  -0.915 |
| **Sex(F)** | Estimate  CI  t value | 0.404***  [0.175, 0.633]  3.478 | 0.399***  [0.174, 0.625]  3.498 | 0.421***  [0.195, 0.646]  3.682 | 0.400***  [0.174, 0.625]  3.492 | 0.393***  [0.164, 0.622]  3.383 | 0.410***  [0.184, 0.635]  3.586 | 0.383**  [0.156, 0.610]  3.323 |
| **Education** | Estimate  CI  t value | -0.001  [-0.110, 0.108]  -0.014 | -0.003  [-0.112, 0.106]  -0.058 | -0.006  [-0.114, 0.102]  -0.102 | -0.001  [-0.109, 0.108]  -0.016 | -0.001  [-0.109, 0.108]  -0.014 | -0.006  [-0.115, 0.102]  -0.117 | -0.000  [-0.108, 0.108]  -0.004 |
| **Age** | Estimate  CI  t value | -0.314***  [-0.426, -0.201]  -5.480 | -0.319***  [-0.434, -0.204]  -5.459 | -0.323***  [-0.435, -0.211]  -5.688 | -0.313***  [-0.426, -0.200]  -5.454 | -0.312***  [-0.423, -0.200]  -5.489 | -0.308***  [-0.420, -0.196]  -5.434 | -0.290***  [-0.408, -0.171]  -4.820 |
| **ApoE4(+)** | Estimate  CI  t value | -0.065  [-0.331, 0.202]  -0.479 | -0.077  [-0.337, 0.184]  -0.581 | -0.073  [-0.319, 0.173]  -0.587 | -0.053  [-0.301, 0.194]  -0.426 | -0.052  [-0.299, 0.194]  -0.419 | -0.097  [-0.354, 0.160]  -0.743 | -0.056  [-0.302, 0.190]  -0.448 |
| **ATA+T-** | Estimate  CI  t value | -0.240  [-0.553, 0.072]  -1.517 | -0.235  [-0.547, 0.077]  -1.486 | -0.196  [-0.510, 0.118]  -1.232 | -0.245  [-0.556, 0.066]  -1.553 | -0.239  [-0.549, 0.071]  -1.518 | -0.221  [-0.532, 0.091]  -1.397 | -0.280  [-0.596, 0.035]  -1.754 |
| **ATA-T+** | Estimate  CI  t value | -0.214  [-0.570, 0.142]  -1.183 | -0.214  [-0.548, 0.120]  -1.262 | -0.341  [-0.710, 0.029]  -1.819 | -0.199  [-0.530, 0.132]  -1.184 | -0.192  [-0.524, 0.140]  -1.139 | -0.290  [-0.652, 0.073]  -1.575 | -0.148  [-0.490, 0.193]  -0.856 |
| **ATA+T+** | Estimate  CI  t value | -0.285  [-0.611, 0.041]  -1.722 | -0.290  [-0.603, 0.024]  -1.823 | -0.364*  [-0.687, -0.040]  -2.215 | -0.277  [-0.585, 0.032]  -1.767 | -0.271  [-0.579, 0.037]  -1.733 | -0.355*  [-0.692, -0.019]  -2.084 | -0.217  [-0.538, 0.104]  -1.332 |
| **ATA+T-:time** | Estimate  CI  t value | -0.002  [-0.098, 0.093]  -0.050 | -0.003  [-0.099, 0.093]  -0.056 | -0.005  [-0.103, 0.092]  -0.110 | -0.005  [-0.100, 0.090]  -0.112 | 0.006  [-0.090, 0.102]  0.123 | -0.001  [-0.097, 0.096]  -0.018 | -0.014  [-0.110, 0.082]  -0.287 |
| **ATA-T+:time** | Estimate  CI  t value | -0.021  [-0.136, 0.095]  -0.354 | -0.028  [-0.138, 0.082]  -0.500 | -0.025  [-0.147, 0.097]  -0.405 | -0.029  [-0.137, 0.078]  -0.536 | -0.023  [-0.132, 0.086]  -0.415 | -0.043  [-0.160, 0.075]  -0.716 | -0.012  [-0.123, 0.099]  -0.214 |
| **ATA+T+:time** | Estimate  CI  t value | -0.094  [-0.205, 0.017]  -1.676 | -0.098  [-0.204, 0.007]  -1.843 | -0.104  [-0.212, 0.003]  -1.922 | -0.112*  [-0.211, -0.013]  -2.227 | -0.102*  [-0.203, -0.002]  -2.015 | -0.120*  [-0.231, -0.009]  -2.127 | -0.083  [-0.188, 0.023]  -1.547 |

*p<0.05, ** p<0.01, ***p<0.001

**Supplementary Table 9** Results of Mixed Effect Models for the Marker Panel

| **Hippocampus** | | | | | | | | | | | |
| --- | --- | --- | --- | --- | --- | --- | --- | --- | --- | --- | --- |
|  | | **FABP3** | **MIF** | **AXL** | **C4** | **Factor B** | **Factor H** | **MCP1** | **IP10** | **Il6** | **IL18** |
| **Intercept** | Estimate  CI  t value | 0.145  [-0.074, 0.364]  1.301 | 0.186  [-0.033, 0.404]  1.677 | 0.179  [-0.038, 0.396]  1.628 | 0.186  [-0.031, 0.402]  1.693 | 0.186  [-0.028, 0.401]  1.713 | 0.180  [-0.036, 0.395]  1.641 | 0.187  [-0.029, 0.404]  1.706 | 0.183  [-0.034, 0.400]  1.661 | 0.182  [-0.034, 0.398]  1.659 | 0.199  [-0.017, 0.415]  1.819 |
| **Marker** | Estimate  CI  t value | -0.104  [-0.225, 0.018]  -1.682 | 0.002  [-0.104, 0.108]  0.040 | 0.044  [-0.060, 0.148]  0.828 | -0.039  [-0.133, 0.056]  -0.809 | -0.090*  [-0.178, -0.003]  -2.030 | -0.071  [-0.161, 0.019]  -1.553 | -0.007  [-0.091, 0.077]  -0.170 | -0.025  [-0.108, 0.057]  -0.604 | 0.014  [-0.064, 0.093]  0.354 | -0.081  [-0.172, 0.010]  -1.750 |
| **Time** | Estimate  CI  t value | -0.029**  [-0.051, -0.007]  -2.643 | -0.020  [-0.042, 0.001]  -1.836 | -0.019  [-0.040, 0.002]  -1.747 | -0.018  [-0.039, 0.004]  -1.613 | -0.019  [-0.040, 0.003]  -1.721 | -0.021  [-0.043, 0.000]  -1.962 | -0.018  [-0.039, 0.003]  -1.656 | -0.019  [-0.040, 0.002]  -1.784 | -0.020  [-0.041, 0.001]  -1.870 | -0.020  [-0.041, 0.002]  -1.829 |
| **Marker:time** | Estimate^1^  CI  t value | **-0.020****  **[-0.034, -0.007]**  **-2.951** | -0.004  [-0.017, 0.009]  -0.610 | -0.000  [-0.013, 0.013]  -0.059 | 0.003  [-0.008, 0.014]  0.509 | -0.000  [-0.011, 0.011]  -0.037 | -0.007  [-0.018, 0.004]  -1.261 | 0.004  [-0.007, 0.015]  0.698 | -0.006  [-0.018, 0.006]  -1.043 | -0.011  [-0.022, 0.001]  -1.760 | -0.005  [-0.017, 0.006]  -0.910 |
| **AD** | Estimate  CI  t value | -1.621***  [-2.002, -1.239]  -8.375 | -1.678***  [-2.060, -1.296]  -8.661 | -1.653***  [-2.038, -1.269]  -8.481 | -1.676***  [-2.056, -1.296]  -8.700 | -1.680***  [-2.056, -1.303]  -8.802 | -1.673***  [-2.051, -1.295]  -8.726 | -1.678***  [-2.060, -1.297]  -8.673 | -1.683***  [-2.063, -1.303]  -8.732 | -1.684***  [-2.064, -1.303]  -8.713 | -1.693***  [-2.071, -1.315]  -8.829 |
| **MCI** | Estimate  CI  t value | -0.679***  [-0.937, -0.421]  -5.190 | -0.681***  [-0.941, -0.421]  -5.166 | -0.654***  [-0.921, -0.388]  -4.834 | -0.678***  [-0.938, -0.419]  -5.149 | -0.663***  [-0.920, -0.405]  -5.069 | -0.670***  [-0.928, -0.411]  -5.104 | -0.681***  [-0.941, -0.421]  -5.164 | -0.682***  [-0.942, -0.422]  -5.179 | -0.688***  [-0.951, -0.425]  -5.155 | -0.672***  [-0.930, -0.414]  -5.128 |
| **SCD** | Estimate  CI  t value | -0.235*  [-0.450, -0.019]  -2.141 | -0.227*  [-0.443, -0.010]  -2.062 | -0.212  [-0.431, 0.008]  -1.901 | -0.224*  [-0.440, -0.007]  -2.034 | -0.210  [-0.425, 0.005]  -1.928 | -0.212  [-0.428, 0.004]  -1.931 | -0.228*  [-0.444, -0.011]  -2.072 | -0.220*  [-0.438, -0.002]  -1.992 | -0.226*  [-0.443, -0.010]  -2.061 | -0.234*  [-0.449, -0.018]  -2.136 |
| **AD:time** | Estimate  CI  t value | -0.118***  [-0.170, -0.067]  -4.524 | -0.135***  [-0.187, -0.084]  -5.175 | -0.135***  [-0.187, -0.083]  -5.110 | -0.135***  [-0.187, -0.084]  -5.169 | -0.134***  [-0.186, -0.083]  -5.138 | -0.134***  [-0.185, -0.083]  -5.142 | -0.137***  [-0.188, -0.085]  -5.193 | -0.135***  [-0.186, -0.083]  -5.162 | -0.129***  [-0.180, -0.077]  -4.937 | -0.134***  [-0.186, -0.083]  -5.143 |
| **MCI:time** | Estimate  CI  t value | -0.068***  [-0.101, -0.034]  -4.013 | -0.069***  [-0.103, -0.035]  -4.025 | -0.069***  [-0.104, -0.034]  -3.930 | -0.070***  [-0.104, -0.036]  -4.044 | -0.069***  [-0.103, -0.035]  -3.992 | -0.068***  [-0.102, -0.034]  -3.947 | -0.069***  [-0.103, -0.035]  -4.027 | -0.069***  [-0.103, -0.035]  -4.009 | -0.062***  [-0.097, -0.028]  -3.575 | -0.067***  [-0.101, -0.033]  -3.878 |
| **SCD:time** | Estimate  CI  t value | -0.021  [-0.047, 0.004]  -1.641 | -0.022  [-0.048, 0.004]  -1.646 | -0.023  [-0.049, 0.004]  -1.677 | -0.023  [-0.049, 0.003]  -1.721 | -0.022  [-0.049, 0.004]  -1.688 | -0.020  [-0.046, 0.006]  -1.502 | -0.023  [-0.049, 0.003]  -1.739 | -0.021  [-0.047, 0.006]  -1.557 | -0.020  [-0.046, 0.006]  -1.509 | -0.022  [-0.048, 0.004]  -1.651 |
| **Sex(F)** | Estimate  CI  t value | 0.440***  [0.249, 0.631]  4.543 | 0.459***  [0.271, 0.647]  4.819 | 0.469***  [0.280, 0.658]  4.891 | 0.443***  [0.253, 0.633]  4.593 | 0.429***  [0.242, 0.617]  4.505 | 0.436***  [0.246, 0.627]  4.519 | 0.457***  [0.269, 0.645]  4.795 | 0.462***  [0.273, 0.650]  4.823 | 0.464***  [0.276, 0.652]  4.869 | 0.427***  [0.237, 0.618]  4.414 |
| **Education** | Estimate  CI  t value | 0.015  [-0.076, 0.105]  0.321 | 0.011  [-0.079, 0.101]  0.240 | 0.008  [-0.083, 0.098]  0.168 | 0.009  [-0.082, 0.099]  0.190 | 0.005  [-0.085, 0.094]  0.105 | 0.008  [-0.082, 0.099]  0.184 | 0.010  [-0.081, 0.100]  0.215 | 0.012  [-0.078, 0.103]  0.272 | 0.012  [-0.078, 0.102]  0.260 | 0.016  [-0.074, 0.106]  0.346 |
| **Age** | Estimate  CI  t value | -0.342***  [-0.436, -0.248]  -7.171 | -0.348***  [-0.442, -0.254]  -7.309 | -0.346***  [-0.439, -0.253]  -7.341 | -0.342***  [-0.435, -0.248]  -7.220 | -0.355***  [-0.447, -0.262]  -7.557 | -0.345***  [-0.438, -0.252]  -7.312 | -0.344***  [-0.438, -0.250]  -7.191 | -0.346***  [-0.439, -0.252]  -7.308 | -0.347***  [-0.441, -0.254]  -7.359 | -0.335***  [-0.429, -0.241]  -7.043 |
| **ApoE4(+)** | Estimate  CI  t value | -0.030  [-0.251, 0.191]  -0.268 | -0.075  [-0.281, 0.131]  -0.718 | -0.082  [-0.288, 0.124]  -0.785 | -0.071  [-0.275, 0.134]  -0.679 | -0.069  [-0.272, 0.135]  -0.665 | -0.063  [-0.269, 0.142]  -0.610 | -0.071  [-0.277, 0.135]  -0.679 | -0.074  [-0.279, 0.131]  -0.712 | -0.081  [-0.287, 0.125]  -0.773 | -0.055  [-0.261, 0.150]  -0.531 |
| **ATA+T-** | Estimate  CI  t value | -0.065  [-0.331, 0.201]  -0.480 | -0.049  [-0.318, 0.219]  -0.361 | -0.026  [-0.298, 0.246]  -0.189 | -0.045  [-0.311, 0.222]  -0.330 | -0.036  [-0.300, 0.227]  -0.272 | -0.042  [-0.306, 0.223]  -0.309 | -0.053  [-0.319, 0.214]  -0.390 | -0.055  [-0.321, 0.211]  -0.405 | -0.049  [-0.315, 0.216]  -0.366 | -0.060  [-0.325, 0.205]  -0.447 |
| **ATA-T+** | Estimate  CI  t value | -0.036  [-0.337, 0.265]  -0.236 | -0.129  [-0.437, 0.178]  -0.829 | -0.168  [-0.468, 0.131]  -1.108 | -0.117  [-0.401, 0.168]  -0.808 | -0.119  [-0.399, 0.162]  -0.833 | -0.103  [-0.386, 0.181]  -0.715 | -0.126  [-0.410, 0.159]  -0.871 | -0.131  [-0.415, 0.152]  -0.912 | -0.123  [-0.407, 0.160]  -0.857 | -0.121  [-0.403, 0.161]  -0.848 |
| **ATA+T+** | Estimate  CI  t value | -0.241  [-0.516, 0.033]  -1.735 | -0.329*  [-0.599, -0.059]  -2.405 | -0.359**  [-0.630, -0.087]  -2.607 | -0.321*  [-0.584, -0.058]  -2.408 | -0.327*  [-0.587, -0.068]  -2.485 | -0.318*  [-0.579, -0.057]  -2.405 | -0.332*  [-0.594, -0.069]  -2.492 | -0.325*  [-0.587, -0.062]  -2.441 | -0.317*  [-0.584, -0.051]  -2.348 | -0.334*  [-0.595, -0.073]  -2.525 |
| **ATA+T-:time** | Estimate  CI  t value | -0.003  [-0.034, 0.028]  -0.172 | -0.004  [-0.036, 0.028]  -0.260 | -0.003  [-0.035, 0.029]  -0.191 | -0.004  [-0.036, 0.028]  -0.246 | -0.003  [-0.035, 0.029]  -0.191 | -0.001  [-0.032, 0.031]  -0.045 | -0.003  [-0.035, 0.029]  -0.189 | -0.004  [-0.036, 0.027]  -0.271 | -0.004  [-0.035, 0.027]  -0.247 | -0.002  [-0.034, 0.029]  -0.151 |
| **ATA-T+:time** | Estimate  CI  t value | 0.010  [-0.027, 0.047]  0.522 | -0.005  [-0.044, 0.034]  -0.245 | -0.009  [-0.047, 0.029]  -0.476 | -0.011  [-0.047, 0.025]  -0.597 | -0.010  [-0.046, 0.026]  -0.539 | -0.006  [-0.042, 0.030]  -0.314 | -0.011  [-0.047, 0.025]  -0.601 | -0.010  [-0.046, 0.026]  -0.557 | -0.010  [-0.045, 0.026]  -0.531 | -0.009  [-0.044, 0.027]  -0.469 |
| **ATA+T+:time** | Estimate  CI  t value | -0.068***  [-0.102, -0.033]  -3.825 | -0.087***  [-0.120, -0.053]  -5.078 | -0.089***  [-0.123, -0.056]  -5.218 | -0.090***  [-0.123, -0.058]  -5.490 | -0.090***  [-0.122, -0.057]  -5.469 | -0.087***  [-0.119, -0.054]  -5.271 | -0.090***  [-0.123, -0.058]  -5.504 | -0.089***  [-0.122, -0.057]  -5.464 | -0.094***  [-0.126, -0.061]  -5.714 | -0.089***  [-0.121, -0.057]  -5.443 |

1pFDR = 0.025. *p<0.05, ** p<0.01, ***p<0.001.

**Supplementary Table 10** Results of Mixed Effect Models for the Marker Panel

| **Basal Forebrain** | | | | | | | | | | | |
| --- | --- | --- | --- | --- | --- | --- | --- | --- | --- | --- | --- |
|  | | **FABP3** | **MIF** | **AXL** | **C4** | **Factor B** | **Factor H** | **MCP1** | **IP10** | **Il6** | **IL18** |
| **Intercept** | Estimate  CI  t value | 0.126  [-0.133, 0.385]  0.959 | 0.117  [-0.140, 0.375]  0.898 | 0.124  [-0.132, 0.379]  0.956 | 0.129  [-0.126, 0.384]  0.999 | 0.125  [-0.130, 0.379]  0.967 | 0.124  [-0.130, 0.379]  0.963 | 0.126  [-0.130, 0.381]  0.971 | 0.121  [-0.134, 0.377]  0.937 | 0.123  [-0.131, 0.378]  0.957 | 0.136  [-0.120, 0.392]  1.049 |
| **Marker** | Estimate  CI  t value | -0.003  [-0.146, 0.141]  -0.040 | -0.042  [-0.165, 0.082]  -0.664 | 0.023  [-0.098, 0.145]  0.377 | 0.030  [-0.080, 0.140]  0.537 | -0.043  [-0.146, 0.060]  -0.818 | -0.021  [-0.126, 0.085]  -0.385 | 0.006  [-0.093, 0.104]  0.115 | -0.034  [-0.132, 0.063]  -0.691 | 0.041  [-0.052, 0.134]  0.864 | -0.051  [-0.158, 0.057]  -0.929 |
| **Time** | Estimate  CI  t value | 0.059  [-0.010, 0.128]  1.690 | 0.052  [-0.013, 0.117]  1.580 | 0.068*  [0.003, 0.133]  2.062 | 0.064  [-0.002, 0.131]  1.908 | 0.061  [-0.004, 0.127]  1.840 | 0.058  [-0.008, 0.125]  1.738 | 0.063  [-0.002, 0.128]  1.899 | 0.067*  [0.002, 0.132]  2.035 | 0.066*  [0.001, 0.132]  2.002 | 0.063  [-0.002, 0.129]  1.903 |
| **Marker:time** | Estimate  CI  t value | -0.017  [-0.060, 0.026]  -0.782 | -0.049*  [-0.087, -0.010]  -2.504 | -0.007  [-0.047, 0.032]  -0.366 | -0.010  [-0.045, 0.024]  -0.602 | -0.024  [-0.058, 0.010]  -1.407 | -0.023  [-0.056, 0.011]  -1.332 | -0.025  [-0.058, 0.008]  -1.477 | -0.021  [-0.057, 0.015]  -1.158 | -0.016  [-0.054, 0.022]  -0.850 | -0.019  [-0.053, 0.016]  -1.056 |
| **AD** | Estimate  CI  t value | -1.011***  [-1.463, -0.559]  -4.410 | -1.028***  [-1.478, -0.578]  -4.506 | -1.001***  [-1.453, -0.548]  -4.357 | -1.018***  [-1.465, -0.571]  -4.491 | -1.017***  [-1.463, -0.571]  -4.497 | -1.015***  [-1.462, -0.568]  -4.480 | -1.016***  [-1.465, -0.566]  -4.456 | -1.019***  [-1.466, -0.572]  -4.493 | -1.031***  [-1.479, -0.584]  -4.541 | -1.022***  [-1.469, -0.575]  -4.509 |
| **MCI** | Estimate  CI  t value | -0.614***  [-0.919, -0.309]  -3.973 | -0.620***  [-0.925, -0.315]  -4.009 | -0.599***  [-0.913, -0.286]  -3.769 | -0.616***  [-0.921, -0.312]  -3.989 | -0.605***  [-0.910, -0.301]  -3.918 | -0.613***  [-0.918, -0.308]  -3.968 | -0.617***  [-0.922, -0.312]  -3.986 | -0.613***  [-0.917, -0.309]  -3.971 | -0.637***  [-0.945, -0.328]  -4.071 | -0.607***  [-0.911, -0.302]  -3.930 |
| **SCD** | Estimate  CI  t value | -0.090  [-0.343, 0.164]  -0.700 | -0.088  [-0.341, 0.165]  -0.685 | -0.083  [-0.339, 0.173]  -0.638 | -0.095  [-0.347, 0.158]  -0.738 | -0.082  [-0.335, 0.171]  -0.640 | -0.085  [-0.338, 0.168]  -0.662 | -0.090  [-0.343, 0.163]  -0.701 | -0.081  [-0.335, 0.172]  -0.633 | -0.092  [-0.344, 0.160]  -0.722 | -0.094  [-0.347, 0.158]  -0.737 |
| **AD:time** | Estimate  CI  t value | -0.124  [-0.291, 0.043]  -1.468 | -0.153  [-0.313, 0.008]  -1.877 | -0.141  [-0.305, 0.023]  -1.694 | -0.135  [-0.299, 0.029]  -1.627 | -0.141  [-0.304, 0.022]  -1.707 | -0.137  [-0.300, 0.025]  -1.666 | -0.127  [-0.290, 0.036]  -1.530 | -0.139  [-0.301, 0.024]  -1.678 | -0.130  [-0.295, 0.034]  -1.565 | -0.135  [-0.298, 0.028]  -1.630 |
| **MCI:time** | Estimate  CI  t value | -0.095  [-0.201, 0.010]  -1.792 | -0.101  [-0.204, 0.002]  -1.934 | -0.100  [-0.208, 0.007]  -1.848 | -0.093  [-0.199, 0.013]  -1.732 | -0.091  [-0.196, 0.014]  -1.706 | -0.093  [-0.198, 0.012]  -1.755 | -0.094  [-0.199, 0.010]  -1.780 | -0.096  [-0.201, 0.008]  -1.813 | -0.087  [-0.195, 0.021]  -1.597 | -0.088  [-0.194, 0.018]  -1.639 |
| **SCD:time** | Estimate  CI  t value | -0.040  [-0.120, 0.039]  -1.007 | -0.035  [-0.113, 0.042]  -0.901 | -0.044  [-0.125, 0.036]  -1.086 | -0.040  [-0.120, 0.040]  -0.993 | -0.036  [-0.115, 0.043]  -0.896 | -0.033  [-0.113, 0.047]  -0.823 | -0.038  [-0.117, 0.041]  -0.942 | -0.036  [-0.116, 0.043]  -0.901 | -0.038  [-0.118, 0.042]  -0.945 | -0.039  [-0.118, 0.041]  -0.963 |
| **Sex(F)** | Estimate  CI  t value | 0.400***  [0.170, 0.629]  3.434 | 0.396***  [0.171, 0.622]  3.462 | 0.405***  [0.179, 0.632]  3.522 | 0.411***  [0.182, 0.639]  3.547 | 0.388***  [0.160, 0.615]  3.360 | 0.393***  [0.164, 0.622]  3.383 | 0.401***  [0.175, 0.626]  3.499 | 0.407***  [0.180, 0.633]  3.542 | 0.407***  [0.182, 0.632]  3.560 | 0.379**  [0.149, 0.609]  3.244 |
| **Education** | Estimate  CI  t value | 0.000  [-0.109, 0.109]  0.003 | -0.000  [-0.109, 0.109]  -0.000 | -0.002  [-0.110, 0.107]  -0.027 | 0.002  [-0.107, 0.110]  0.029 | -0.002  [-0.111, 0.106]  -0.041 | -0.001  [-0.109, 0.108]  -0.012 | 0.000  [-0.108, 0.109]  0.005 | 0.001  [-0.107, 0.110]  0.025 | 0.002  [-0.106, 0.110]  0.036 | 0.003  [-0.106, 0.111]  0.050 |
| **Age** | Estimate  CI  t value | -0.312***  [-0.425, -0.199]  -5.441 | -0.309***  [-0.422, -0.196]  -5.397 | -0.312***  [-0.424, -0.200]  -5.495 | -0.315***  [-0.427, -0.203]  -5.536 | -0.316***  [-0.428, -0.204]  -5.553 | -0.311***  [-0.423, -0.199]  -5.478 | -0.314***  [-0.427, -0.201]  -5.465 | -0.311***  [-0.423, -0.199]  -5.479 | -0.314***  [-0.426, -0.202]  -5.542 | -0.305***  [-0.418, -0.191]  -5.304 |
| **ApoE4(+)** | Estimate  CI  t value | -0.054  [-0.320, 0.212]  -0.402 | -0.050  [-0.298, 0.198]  -0.398 | -0.058  [-0.306, 0.189]  -0.465 | -0.055  [-0.302, 0.191]  -0.444 | -0.052  [-0.298, 0.194]  -0.420 | -0.052  [-0.299, 0.195]  -0.417 | -0.055  [-0.302, 0.192]  -0.439 | -0.053  [-0.299, 0.193]  -0.426 | -0.066  [-0.313, 0.181]  -0.527 | -0.040  [-0.288, 0.208]  -0.316 |
| **ATA+T-** | Estimate  CI  t value | -0.244  [-0.557, 0.068]  -1.541 | -0.260  [-0.574, 0.054]  -1.633 | -0.231  [-0.549, 0.086]  -1.435 | -0.249  [-0.560, 0.061]  -1.582 | -0.234  [-0.544, 0.076]  -1.489 | -0.239  [-0.549, 0.071]  -1.522 | -0.242  [-0.553, 0.069]  -1.535 | -0.250  [-0.560, 0.060]  -1.589 | -0.243  [-0.553, 0.066]  -1.548 | -0.250  [-0.560, 0.060]  -1.590 |
| **ATA-T+** | Estimate  CI  t value | -0.200  [-0.554, 0.154]  -1.113 | -0.156  [-0.515, 0.203]  -0.858 | -0.225  [-0.575, 0.124]  -1.271 | -0.211  [-0.542, 0.121]  -1.251 | -0.193  [-0.523, 0.137]  -1.153 | -0.191  [-0.524, 0.141]  -1.136 | -0.203  [-0.535, 0.129]  -1.205 | -0.208  [-0.538, 0.123]  -1.239 | -0.194  [-0.524, 0.136]  -1.160 | -0.197  [-0.527, 0.133]  -1.175 |
| **ATA+T+** | Estimate  CI  t value | -0.273  [-0.597, 0.050]  -1.664 | -0.250  [-0.567, 0.067]  -1.554 | -0.293  [-0.612, 0.025]  -1.814 | -0.285  [-0.593, 0.024]  -1.818 | -0.273  [-0.580, 0.035]  -1.750 | -0.270  [-0.578, 0.038]  -1.730 | -0.275  [-0.583, 0.034]  -1.755 | -0.274  [-0.582, 0.034]  -1.754 | -0.249  [-0.562, 0.063]  -1.573 | -0.282  [-0.589, 0.026]  -1.806 |
| **ATA+T-:time** | Estimate  CI  t value | -0.002  [-0.098, 0.094]  -0.043 | -0.019  [-0.113, 0.076]  -0.391 | -0.006  [-0.103, 0.092]  -0.119 | 0.001  [-0.096, 0.098]  0.021 | 0.006  [-0.090, 0.103]  0.133 | 0.006  [-0.090, 0.102]  0.118 | -0.002  [-0.097, 0.093]  -0.038 | -0.008  [-0.104, 0.088]  -0.161 | -0.003  [-0.099, 0.092]  -0.071 | 0.000  [-0.095, 0.096]  0.008 |
| **ATA-T+:time** | Estimate  CI  t value | -0.020  [-0.135, 0.095]  -0.339 | 0.019  [-0.096, 0.133]  0.322 | -0.029  [-0.143, 0.085]  -0.498 | -0.031  [-0.140, 0.078]  -0.564 | -0.027  [-0.135, 0.082]  -0.484 | -0.022  [-0.131, 0.087]  -0.402 | -0.027  [-0.135, 0.081]  -0.494 | -0.038  [-0.145, 0.070]  -0.690 | -0.036  [-0.144, 0.072]  -0.663 | -0.030  [-0.138, 0.078]  -0.556 |
| **ATA+T+:time** | Estimate  CI  t value | -0.093  [-0.203, 0.017]  -1.665 | -0.076  [-0.177, 0.026]  -1.471 | -0.106*  [-0.211, -0.002]  -2.006 | -0.109*  [-0.209, -0.008]  -2.140 | -0.107*  [-0.207, -0.007]  -2.121 | -0.101*  [-0.202, -0.001]  -1.989 | -0.108*  [-0.208, -0.009]  -2.153 | -0.112*  [-0.211, -0.012]  -2.221 | -0.118*  [-0.219, -0.017]  -2.315 | -0.109*  [-0.209, -0.010]  -2.167 |

*p<0.05, ** p<0.01, ***p<0.001

**Supplementary Table 11** Results of Mediation Models for Hippocampus

| Path | **Synaptic** | **YKL40** |
| --- | --- | --- |
| **Marker ~ A^+^T^-^** | -0.270***  -0.107  [-0.364, -0.177] | -0.424***  -0.173  [-0.521, -0.325] |
| **Marker ~**  **A^-^T^+^** | 0.867***  0.311  [0.750, 0.983] | 0.663***  0.245  [0.557, 0.767] |
| **Marker ~ A^+^T^+^** | 0.610***  0.284  [0.477, 0.736] | 0.623***  0.298  [0.465, 0.768] |
| **Marker ~ SCD** | -0.181***  -0.091  [-0.269, -0.092] | -0.044  -0.022  [-0.128, 0.045] |
| **Marker ~ MCI** | -0.081  -0.034  [-0.192, 0.040] | -0.101  -0.043  [-0.237, 0.035] |
| **Marker ~ AD** | 0.579***  0.140  [0.373, 0.796] | -0.024  -0.006  [-0.220, 0.196] |
| **Marker ~ age** | 0.028***  0.160  [0.020, 0.035] | 0.054***  0.320  [0.046, 0.063] |
| **Marker ~ sex** | -0.322***  -0.168  [-0.402, -0.250] | -0.208***  -0.112  [-0.292, -0.121] |
| **Marker ~ ApoE4** | 0.678***  0.330  [0.580, 0.776] | -0.025  -0.013  [-0.129, 0.085] |
| **HC slope ~ Marker** | -0.148***  -0.143  [-0.203, -0.095] | -0.160***  -0.150  [-0.216, -0.105] |
| **HC slope ~ SCD** | -0.147***  -0.071  [-0.229, -0.069] | -0.127**  -0.062  [-0.209, -0.051] |
| **HC slope ~ MCI** | -0.648***  -0.260  [-0.765, -0.527] | -0.652***  -0.262  [-0.765, -0.534] |
| **HC slope ~ AD** | -1.353***  -0.316  [-1.542, -1.141] | -1.443***  -0.337  [-1.654, -1.207] |
| **HC slope ~ age** | -0.048***  -0.265  [-0.055, -0.040] | -0.043***  -0.240  [-0.051, -0.035] |
| **HC slope ~ sex** | 0.101*  0.051  [0.025, 0.181] | 0.116**  0.058  [0.040, 0.195] |
| **HC slope ~ ApoE4** | -0.095  -0.045  [-0.196, 0.002] | -0.200***  -0.094  [-0.295, -0.107] |

*p<0.05, ** p<0.01, ***p<0.001


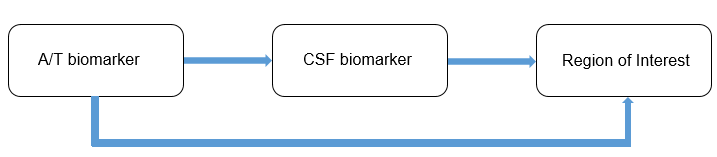


**Supplementary Fig 2** Mediation Diagram
